# Heteroatom-doped carbon dots from medicinal plants as novel biomaterials for as-use biomedical applications in comparison with synthetic drug, zaltoprofen

**DOI:** 10.1038/s41598-024-63700-w

**Published:** 2024-06-07

**Authors:** Sobha Kota, Pradeep Dumpala, Radhika Sajja, Ratnakumari Anantha

**Affiliations:** 1 Department of Chemical Engineering, RVR & JC College of Engineering (A), Guntur, Andhra Pradesh 522019 India; 2 Department of Mechanical Engineering, RVR & JC College of Engineering (A), Guntur, Andhra Pradesh 522019 India

**Keywords:** Medicinal plants, FN-doped carbon dots, Zaltoprofen, Biological functions, Biomedical potential, Biological techniques, Biophysics, Cancer, Cell biology, Microbiology, Plant sciences, Medical research

## Abstract

FN-doped carbon dots were synthesized using powdered leaves of *Moringa oleifera* L./*Chromolaena odorata* L./*Tridax procumbens* L./*Tinospora cordifolia* L./ and *Lantana camara* L., along with a precursor called 4,5-difluoro-1,2-benzenediamine (DFBD) and compared against the drug zaltoprofen derived carbon dots. They were assessed for their optical and structural characteristics using photoluminescence (optimal emission λ of 600 nm), vibrational (FTIR) spectroscopy (characteristic wave numbers of 1156 and 1269 cm^−1^ for C–F), as well as X-ray diffraction (XRD) (highest intensity at 27.56°) and high-resolution transmission electron microscopy (HR-TEM) (particles in the size range of 15–20 nm). Further, field emission scanning electron microscopy (FESEM) / energy dispersive spectroscopy (EDX) indicated FN doping of oval/oblong carbon dots. Membrane protection in percent is found to be 55.3 and 80.4 for FN-CDs and Z-FN-CDs respectively. The DPPH-free radical scavenging activity by FN-CDs was 69.4%, while with Z-FN-CDs, it was 54.2%. When tested on six bacterial strains (three each for gram-positive and gram-negative), the FN-CDs displayed a halo (ZOI) between 9 and 19 mm, whereas the Z-FN-CDs displayed a clearance zone between 9 and 17 mm. The FN-CDs showed significant emission-red-shift effects and demonstrated concentration-dependent biocompatibility and viability in neuroblastoma and beta-TC6-cell lines.

## Introduction

In recent years, advances in nanoscale technologies have led to the use of nanobiomaterials in medical applications. Fluorescent carbon nanoparticles were discovered by serendipity while purifying single-walled carbon nanotubes^1^. Carbon dots have numerous remarkable properties, such as solubility in aqueous and organic solvents, photoluminescence, high fluorescence, and stability^2,3^. CDs are synthesized by either “top-down”/ “step-wise refinement” (arc discharge, laser ablation, and acidic oxidation) or “bottom-up”/inductive (combustion, microwave, hydrothermal, and electrochemistry) approaches from a wide array of substrates^4–6^. The synthesis of carbon dots from plants *Prosopis juliflora*, *Vachellia nilotica*, *Brassica oleracea*, *Centella asiatica*, *Annona squamosa*, *Azadirachta indica*, and *Mentha piperita* has been attempted in recent times for applications in drug delivery, bioimaging, photothermal therapy, angiogenesis; biological potential such as antibacterial/anti-inflammatory/anti-oxidant properties; detection of metal ions (Fe^3+^), antibiotic (metronidazole), quantitation of hypochlorite, carbendazim (fungicide), and assay of dopamine^7–13^. Medicinal plants can be a suitable natural resource for synthesizing CDs with desired properties, by virtue of their bioactive composition. This is exemplified by the fluorescent hydrogel prepared using cationic carbon dots, acrylic acid and pectin, which showed powerful broad-spectrum antibacterial activity, which is 108.5 times more than that of other hydrogels^14^. Wang et al. found that CDs help in the migration of epithelial cells by epithelial-mesenchymal transition, decrease the inflammatory response and granulation tissue area, and thus contribute to the overall wound healing process^15^. Interestingly, carbon dots derived from diverse materials such as sugarcane, industrial waste and natural honey are finding potential applications in nonlinear optical devices, bioimaging, pharmaceutical and therapeutic applications^16,17^.

Heteroatom (nitrogen, sulphur, boron, phosphorus, and gadolinium) “Doping” is a method to enhance the photoluminiscent characteristics of carbon dots^18–24^. Fluorine is the preferred element because of its highest electronegativity and, hence, high absorptivity of adjacent electrons, resulting in the segregation of cationic and anionic charges^25^. The curative efficacy of drugs^26^, chemical firmness of proteins^27^ in biological systems, and phase alienation in polar/aqueous and non-polar/organic environments are all found to increase with grafted fluorine^28,29^. Low amounts of fluorinated polymers bond with one another, promoting a synergistic effect^30^. Methods used to produce F-doped CDs include ultrasonication^31^, solvothermal^32,33^, one-step ring-opening polymerization–dehydrative carbonization^34^, microwave^35^, hydrothermal^36^, and oxidative cutting techniques^37^. The antecedents of fluorine are melamine and ammonium fluoride^31^, 4,5-difluorobenzene-1,2-diamine^32^, tetrafluoroterephthalic acid^33,34^, polyethyleneimine (600 Da) and fluorinated diglycidyl ethers^35^, levofloxacin^36^, and fluorinated graphite^37^ in most cases. Well known applications of fluorine-doped CDs are in the areas of bacterial imaging, bio-imaging of cell lines like HEK 293, B16-F10, and detection of intracellular silver; efficacious gene transfection and biocompatibility in contrast to commercial reagents like lipofectamine 2000 and polyethylenimine (25 kDa), photodynamic therapy, and measurement of 4-nitrophenol^31–37^. Zaltoprofen, classified as a non-steroidal anti-inflammatory drug, is an analgesic propionic acid derivative with potent anti-inflammatory and anti-nociceptive properties. It acts as a cyclooxygenase inhibitor, decreases the generation of prostaglandin E2 (PGE2), and inhibits the bradykinin and lipoxygenase pathways of nociception^38^. However, non-selective inhibition of COX creates adverse effects such as peptic ulcers, platelet dysfunction and nephrotoxicity.

The bioactive phytoconstituents viz. kaempferol, scutellarein, isosakuranetin, rutin, coumaric acid, quercetin, gallic acid, niazinin, luteolin, and berberine of *Moringa oleifera L.*, *Chromolaena odorata* L., *Tridax procumbens L.*, *Tinospora cordifolia L.* and *Lantana camara L.* exhibit antipyretic, anti-inflammatory, antibacterial, hypoglycemic, immunomodulatory, and antitumor properties^39–43^ due to the presence of various bioactive compounds like flavonoids, polyphenols, alkaloids, quinines etc. Table S1 presents the results of 17 tests performed for preliminary phytochemical screening of the chosen plant leaf extracts. Extraction or Isolation of bioactives from these plant organs is possible, but it is tedious, time consuming, and requires carcinogenic solvents. A good substitute for this traditional method is the green synthesis of desired heteroatom-doped CDs from medicinal plant materials without the use of toxic solvents and a minimal purification protocol^44^. Heteroatom-doped CDs are prepared by doping the CDs in the respective precursor-solution(s). The incorporation of fluorine and nitrogen enhances the mechanical stability under repeated dynamic loading and piezoelectric properties in carbon dots that are essential requirements for their effective functioning as integral parts of biomedical devices employed in tissue regeneration and remodeling. Another significant benefit of heteroatom doping is the tuning of photoluminescence in CDs while enhancing their fluorescence^45^. Thus, the fabrication of heteroatom doped CDs from abundantly available medicinal plants with enhanced therapeutic properties in an eco-friendly, low-cost process is highly desirable.

The main medicinal uses of CDs at the moment are being studied by researchers. These uses include antibacterial, antioxidant, and anti-inflammatory properties, inadequacies in which are the primary cause of illnesses and diseases in humans^3,46^. The use of dietary supplements regularly as a part of traditional methods of treatment exposed their harmful effects on vital organs such as the liver, kidney and heart. Consequently, there was an imperative need for the development of alternatives; One such alternative identified was the synthesis of CDs from medicinal plant components, which have fewer accompanying side effects on living systems. Biomass valorization in recent years inspired the present study of FN-CD synthesis from medicinal plants (*Moringa oleifera* L., *Chromolaena odorata* L., *Tridax procumbens* L., *Tinospora cordifolia* L., *Lantana camara*) and the anti-inflammatory drug Zaltoprofen, employing DFBD as the depot for “fluorine”, and subsequent investigation of the optical, antioxidant, anti-inflammatory, and hypoglycemic properties of doped CDs together with their biocompatibility potential. A one-pot solvothermal-assisted green process was adopted for the synthesis of FN-CDs that emitted yellow fluorescence, and their emission wavelength showed a red shift by approximately 50 nm. FN-CDs derived from plant and Z-FN-CDs synthesized from zaltoprofen, demonstrated excellent biocompatibility when tested on neuroblastoma and beta-TC6-cell lines, reinstating their possible use for biomedical applications. At higher concentrations, they can be used as anticancer agents because of their evident antiproliferative and cytotoxic potential. To the best of our knowledge, this is the first FN-CD synthesized using the principles of a green and sustainable approach and taken forward for wound healing and cytotoxicity assessment. The entire synthesis process is fast, repeatable, and could easily be scaled up with excellent photoluminescence and cytocompatibility at lower concentrations, indicating their imminent importance in biomedical applications as therapeutic agents.

## Results

### Synthesis and characterization

**Figure 1:**
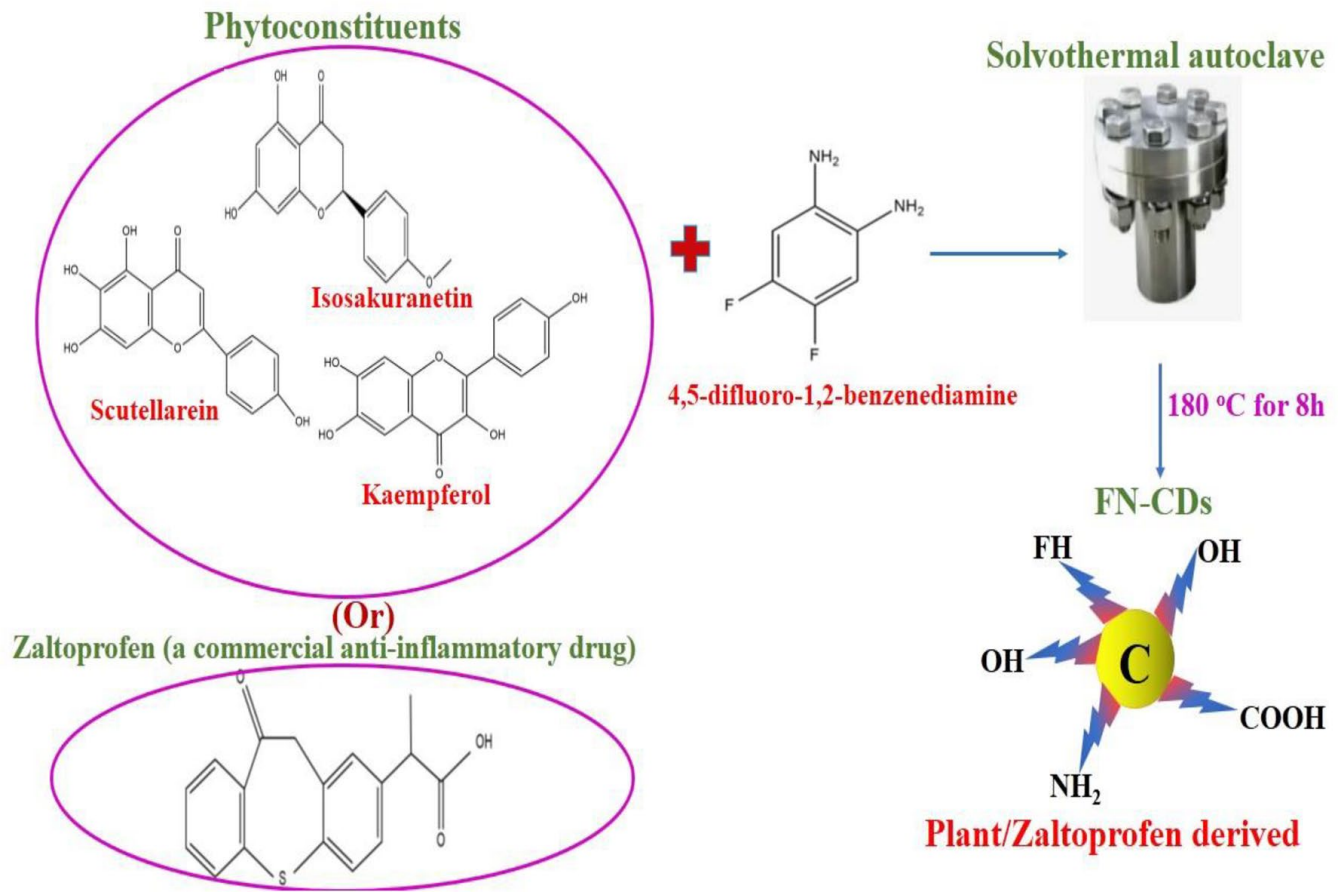
Synthesis of FN-CDs from leaf powders of medicinal plants and Z-FN-CDs from the commercial drug, zaltoprofen.

The biomolecules present in leaf powder are transformed into carbon dots by dehydration and carbonization and doped with F- and N-atoms using DFBD as the precursor agent. Thus, yellow-light-emitting-carbon-dots are formed after the solvothermal-process at 180 °C for 8 h. Similarly, ethyl-alcohol-soluble, zaltoprofen-derived carbon dots doped with F and N atoms are formed with the same precursor and under the same operating conditions as those used in the solvothermal process (Fig. 1).

Knowing the optical band gap from UV absorbance data offers the advantage of facilitating the fabrication of biocompatible and degradable materials in biomedical applications. Energy band gaps between 1 and 2.5 eV, is considered crucial for electronic device manufacturing. Additionally, optical band gaps play a significant role in characterizing carbonaceous materials, aiding in the identification of different structures and their degree of aromatization, which is vital in various biomedical applications. In the present study, Tauc’s method was employed to calculate the direct optical band gap (Fig. S2a) and the same was shown to be between 3.79 and 4.51 eV for FN-CDs, while it was between 3.92 and 4.24 eV for Z-FN-CDs (Fig. S2b).

**Figure 2:**
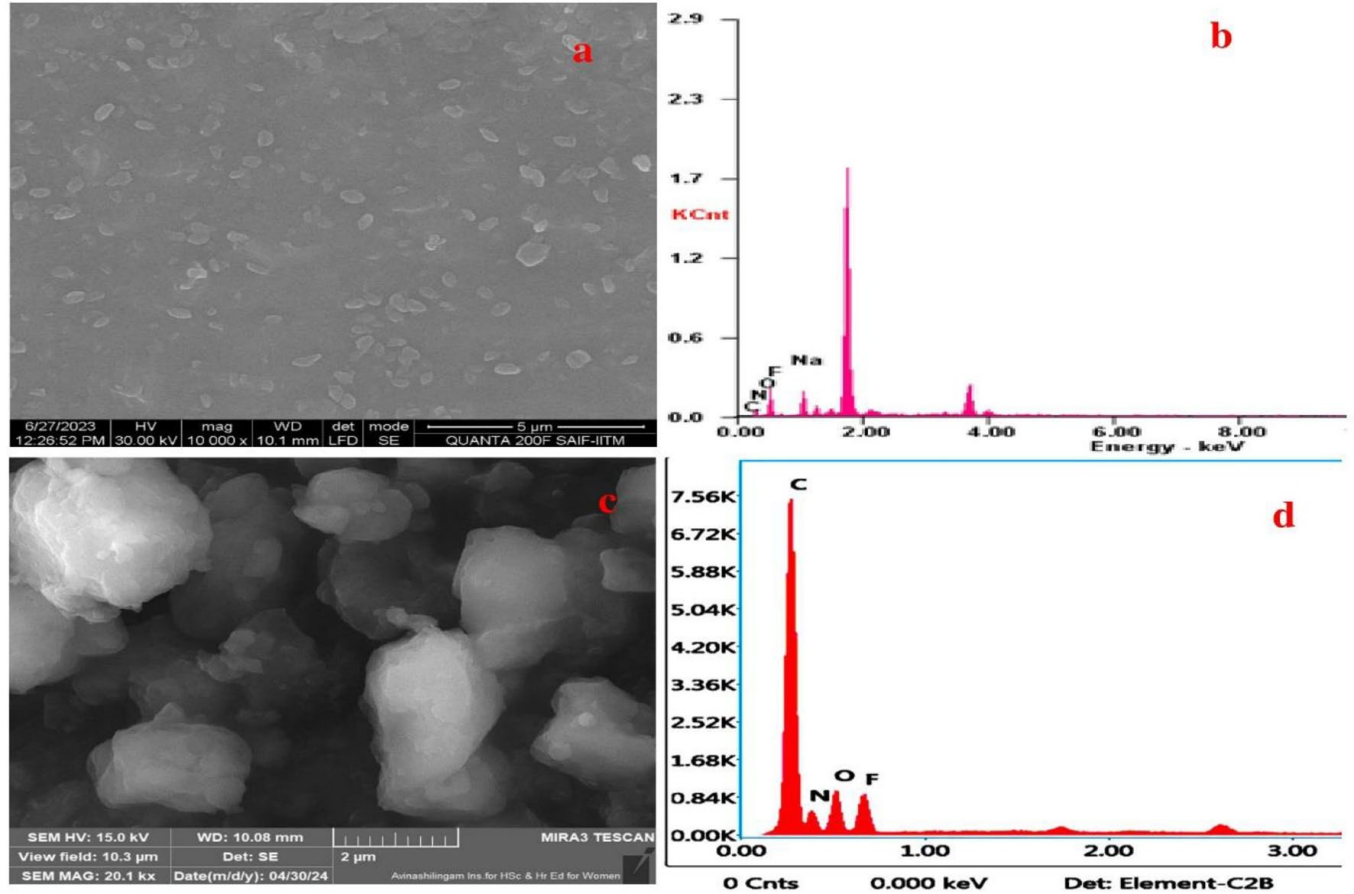
(**a**, **c**) Field emission scanning electron micrographs depicting morphology of FN-CDs & Z-FN-CDs, (**b**, **d**) Elemental composition of FN-CDs & Z-FN-CDs.

**Figure 3:**
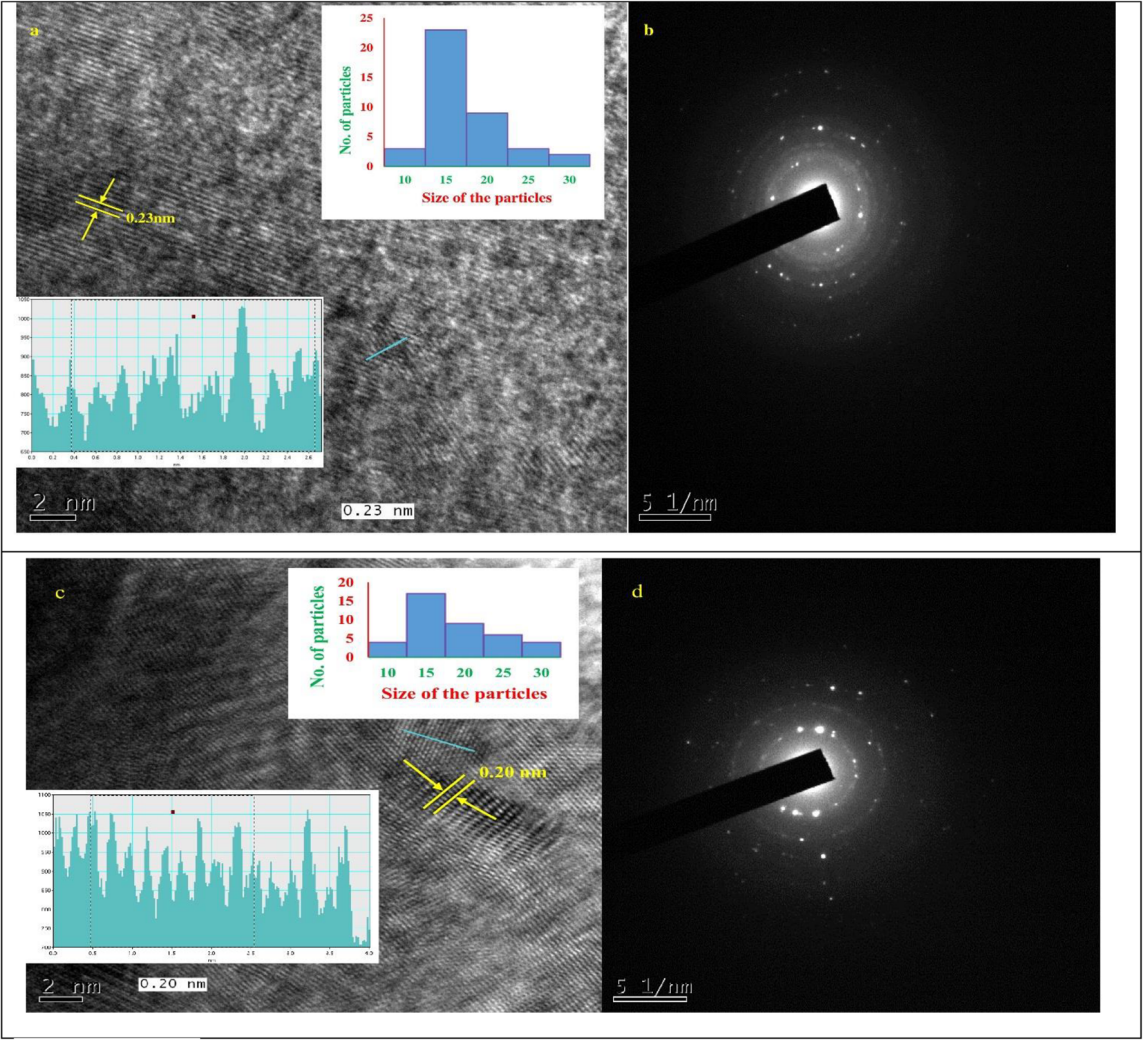
(**a**) High resolution transmission electron microscopic pattern of FN-CDs; (**b**) Z-FN-CDs with inset (i) depicting particle size and distribution, and inset (ii) indicates electron density pattern; (**c**, **d**) represent the selected area electron diffractions of FN-CDs and Z-FN-CDs respectively.

FESEM analysis confirmed the oval/oblong shape of FN-CDs derived from plants and Z-FN-CDs derived from the commercial drug Zaltoprofen (Fig. 2a and c). EDX analyses confirmed surface functional groups and FN doping in the carbon dots, with low intensity signals for fluorine and nitrogen (Fig. 2b, d; Table 1) as compared to carbon and oxygen. Data from the HR-TEM images taken at a resolution of 20 nm and 10 nm are presented in Fig. S1a and b and the images at 2 nm resolution (Fig. 3a and b) were analyzed by image J software for determination of particle sizes. The analyses suggest that the size range of the particles, derived from both the source materials, is predominantly between 15 and 20 nm (Fig. 3a(i) and b(i)). However, significantly higher number of FN-CDs measured 15 nm while for Z-FN-CDs, the difference in the numbers of particles measuring 15 and 20 nm was relatively less. The calculated interplanar distances (Gaton digital micrograph) were similar with 0.23 and 0.2 nm for the FN-CDs and Z-FN-CDs, respectively (Fig. 3a(ii) & b(ii)). The selected area electron diffractograms for the FN-CDs and Z-FN-CDs are presented in Fig. 3c and d.

**Table 1:** Elemental composition of the F- and N-doped carbon dots derived from plants (FN-CDs) and zaltoprofen drug (Z-FN-CDs).

Element	FN-CDs		Z-FN-CDs	
	Weight %	Atomic %	Weight %	Atomic %
C K	22.31	29.59	56.10	62.84
N K	06.64	07.55	15.82	15.2
O K	42.53	42.35	15.72	13.22
F K	05.18	04.34	12.36	8.75
Na K	23.34	16.17	–	–

**Figure 4:**
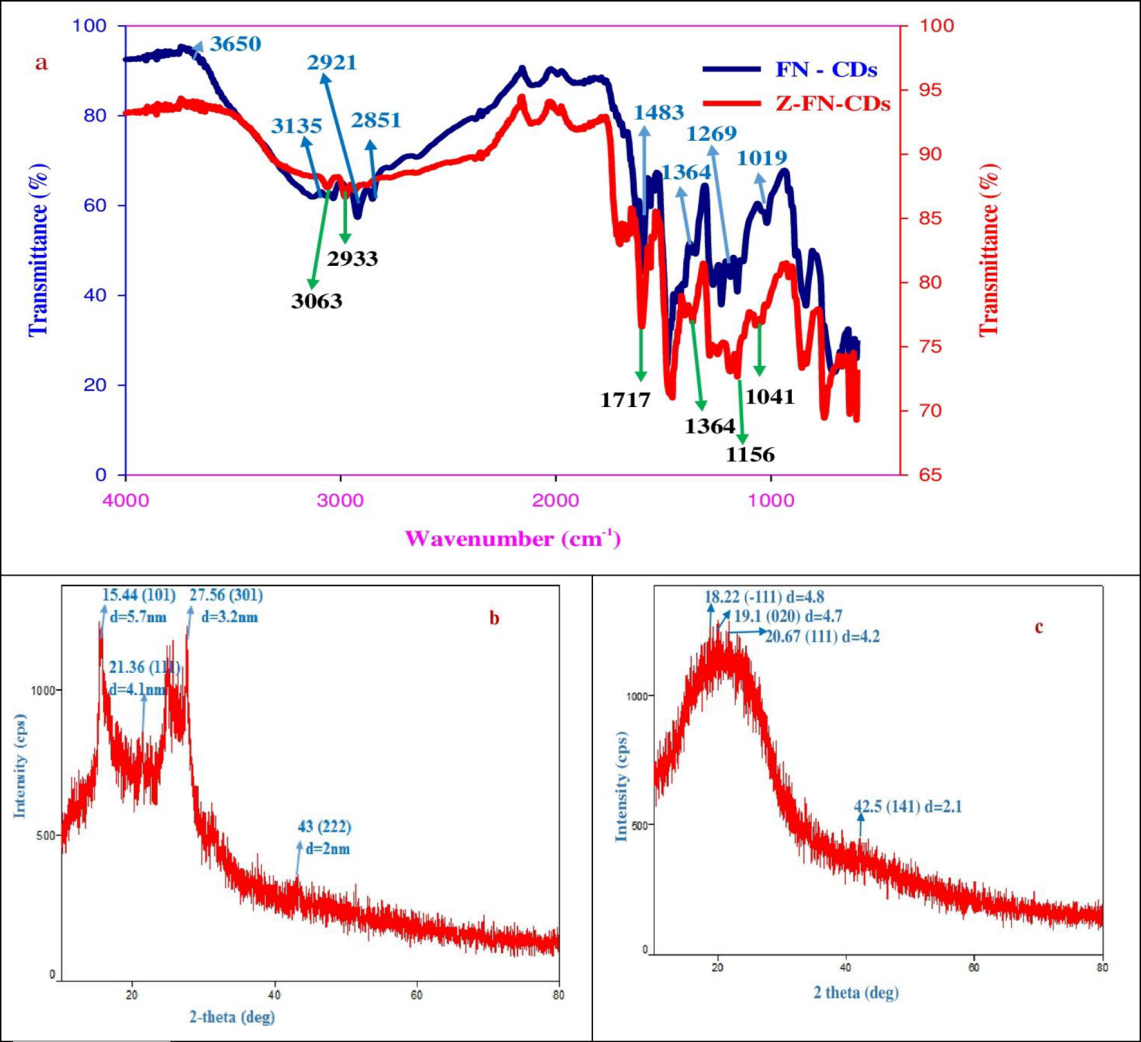
(**a**) FTIR spectra of FN-CDs and Z-FN-CDs with wave numbers corresponding to specific functional groups and associated bond stretching/vibrations; (**b**) X-ray diffractogram of FN-CDs, (**c**) Z-FN-CDs depicting prominent peaks characteristic for carbon dots with markings of 2*θ*, hkl indices and d-spacing as obtained from Match 4 software.

The chemical structure of FN-CDs was characterized using an FTIR-Spectrum (Fig. 4a). The O–H and N–H stretching vibrations are shown by the pulse amplitudes (PAs) at 2851, 2921, 3135, and 3650 cm^−1^, whereas the carboxyl groups are indicated by the PAs at 1735 and 1019 cm^−1^. The existence of a C=O functional group is indicated by the peak at 1650 cm^−1^^47^, and the presence of aromatic rings is indicated by the PAs at 1624 and 1483 cm^−1^, which may be attributed to the vibration of conjugate C=C bonds. The –CH_2_-moiety is indicated by the peak at 1364 cm^−1^. C–F-Bonding is responsible for C–F-Vibrations, particularly the F-aryl-mode at 1156 and 1269 cm^−1^. The Vibrations caused by C–N-Stretching are responsible for the PAs at 1400 and 1200 cm^−1^^48^. Z-FN-CDs’ FTIR-Spectra revealed PAs at 2933 and 3063 cm^−1^, which corresponded to O–H and N–H stretching vibrations; 1717 and 1041 cm^−1^, which were associated with carboxyl groups; and 1647 cm^−1^, which was associated with the C=O functional group^47^. It is possible to attribute the PAs at 1636 and 1473 cm^−1^ to the conjugate C=C-bond-vibrations that characterize aromatic rings. Similar to FN-CDs, the C–F bonding is responsible for the C–F vibrations (F-aryl mode) at 1156 and 1266 cm^−1^. The peak at 1364 cm^−1^ represents the − CH_2_-moiety, and the C–F vibrations (F-aryl mode) at 1156 and 1266 cm^−1^ are attributed to C–F bonding, as in the case of FN-CDs (Fig. 3a).

The X-ray diffractogram of FN-CDs showed a tiny peak at 43°, and strong pulse amplitudes (PAs) at 2*θ*-values of 15.4°, 27. 56°, and 31. 6° demonstrate their crystalline structure (JCPDS 26–1076; Fig. 4b). The presence of functional groups and partial graphitization of the doped carbon dots resulted in less pronounced PAs at 2*θ*-values of 19.1° and 42.5° in the Z-FN-CD-X-ray diffractogram (Fig. 4c). The XRD data were utilized to understand the crystal quality characteristics viz. lattice constant, strain and dislocation density for both FN-CDs and Z-FN-CDs. The calculated values for the three parameters were 10.2 Å, 69.6, 22.9 nm^−2^ respectively for FN-CDs while the same for Z-FN-CDs were 7.4 Å, 152.2 and 62.1 nm^−2^ in the order. In general, decrease in lattice constant means the electrons are more tightly bound to the atom, and hence require more energy to remove, leading to an increased band gap. From the results presented, it is evident that Z-FN-CDs have tightly bound electrons. Further, low strain and dislocation densities for FN-CDs are important indicators of their biomedical applications, predominantly as potential therapeutic agents, drug delivery, bioimaging and biocompatibility.

**Figure 5:**
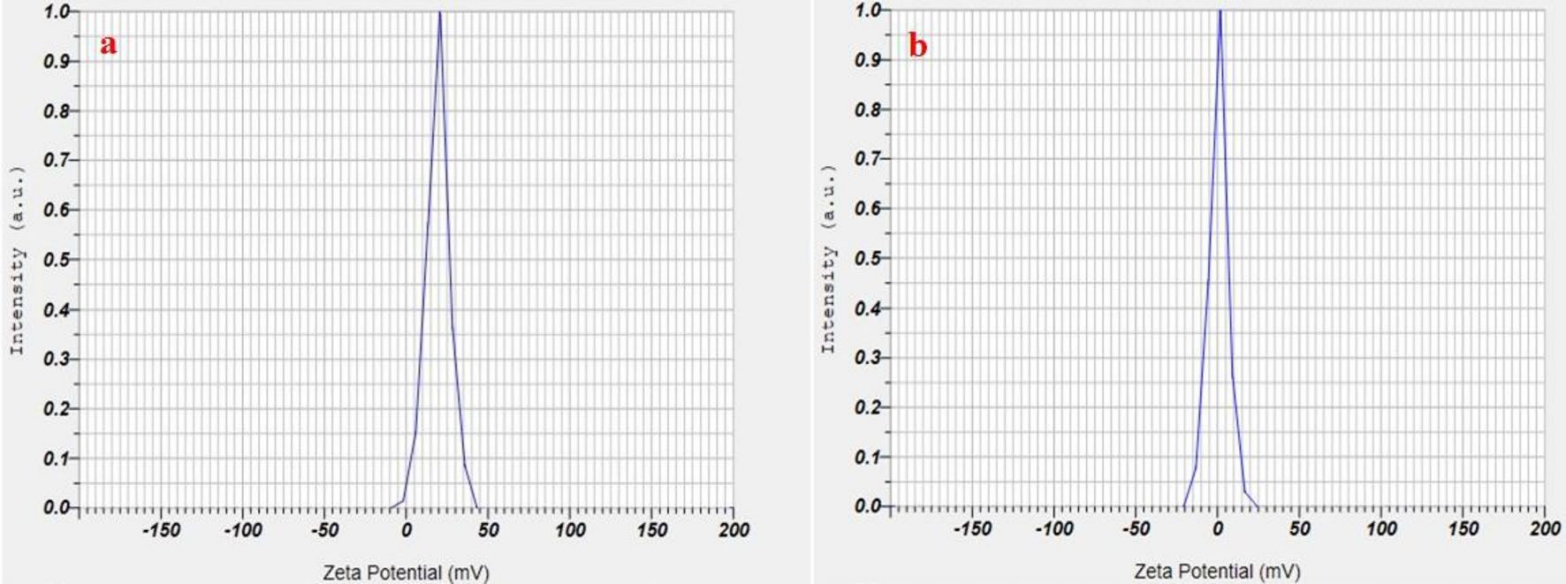
(**a**) Zeta potential distribution of FN-CDs; (**b**) Z-FN-CDs.

An ideal zeta potential value for biomaterial derived carbon dots is positive and it correlates with enhanced biological functions like adherence in wound healing and tissue regeneration because of their biocompatibility and ability to mimic extracellular matrix (ECM), and enhanced stability of emulsions and suspensions due to large positive value induced repulsions between particles. The assessment of zeta potential for FN-CDs showed a sharp peak at 19.4 mV (Fig. 5a), while Z-FN-CDs showed a maximum peak intensity at 0.3 mV (Fig. 5b). These results are suggestive of possible impregnation of FN-CDs in nanofibrous wound dressings and in the formulation of pharmaceutical liquid dosage forms like suspensions and emulsions.

### Optical properties

**Figure 6:**
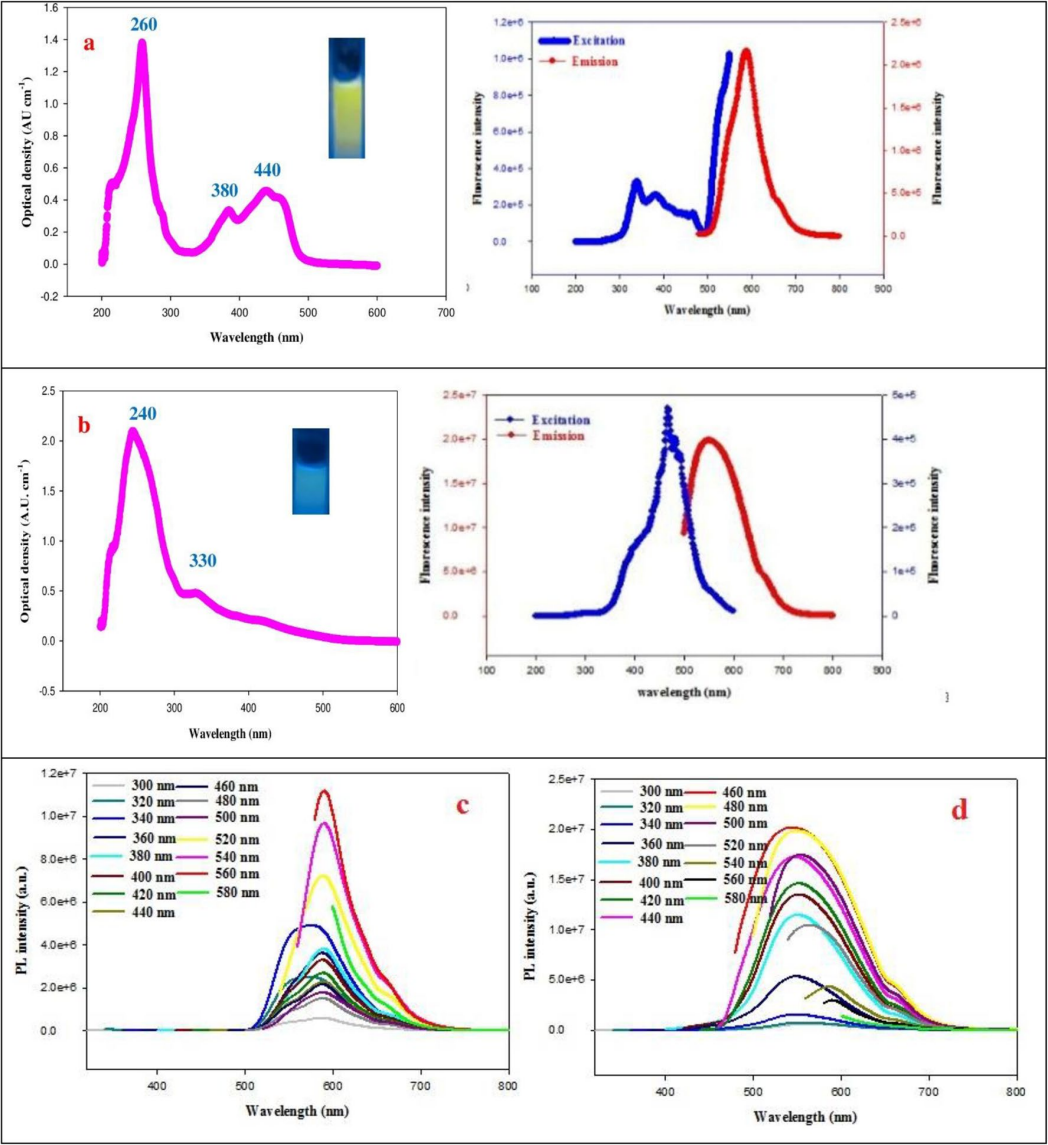
(**a**) UV–vis absorption, fluorescence excitation and emission spectra of FN-CDs and (**b**) Z-FN-CDs; (**c**) excitation-dependent emission fluorescence spectral behavior of FN-CDs and (**d**) Z-FN-CDs.

FN-CDs and Z-FN-CDs’ optical characteristics were examined by UV–vis spectroscopy and photoluminescence experiments^48^. The FN-CDs had three UV–vis-absorption PAs at 260, 380 and 440 nm, as seen in Fig. 6a. These PAs may be associated with the C=N or C=C bonds’ π–π* transition. Furthermore, a wide absorption band spanning from 400 to 500 nm was noted; However, its maximum absorption peak was located at 440 nm and was attributed to the C=O group’s n–π* transition^49,50^. In contrast, two UV–vis-absorption PAs at 240 and 330 nm are present in the Z-FN-CDs (Fig. 6b). Under a UV-Lamp, the FN-CDs and Z-FN-CDs displayed fluorescence (yellow colour) at 365 nm with overlapping excitation and emission spectra (Fig. 6a, b).

By progressively increasing the excitation wavelengths from 300 to 580 nm (at 20 nm intervals), the photoluminescence properties of FN-CDs and Z-FN-CDs were determined. The corresponding emission wavelengths are shown in Fig. 6c and d, respectively. The emission-wavelength of the FN-CDs and Z-FN-CDs ranged from 550 to 600 nm, depending on the excitation-wavelength (300–580 nm). Z-FN-CDs showed the peak at 550 nm against an excitation wavelength of 460 nm, while FN-CDs showed their optimal/maximum emission peak at 600 nm for an excitation wavelength of 560 nm.

## Biological functions

### DPPH scavenging

**Figure 7:**
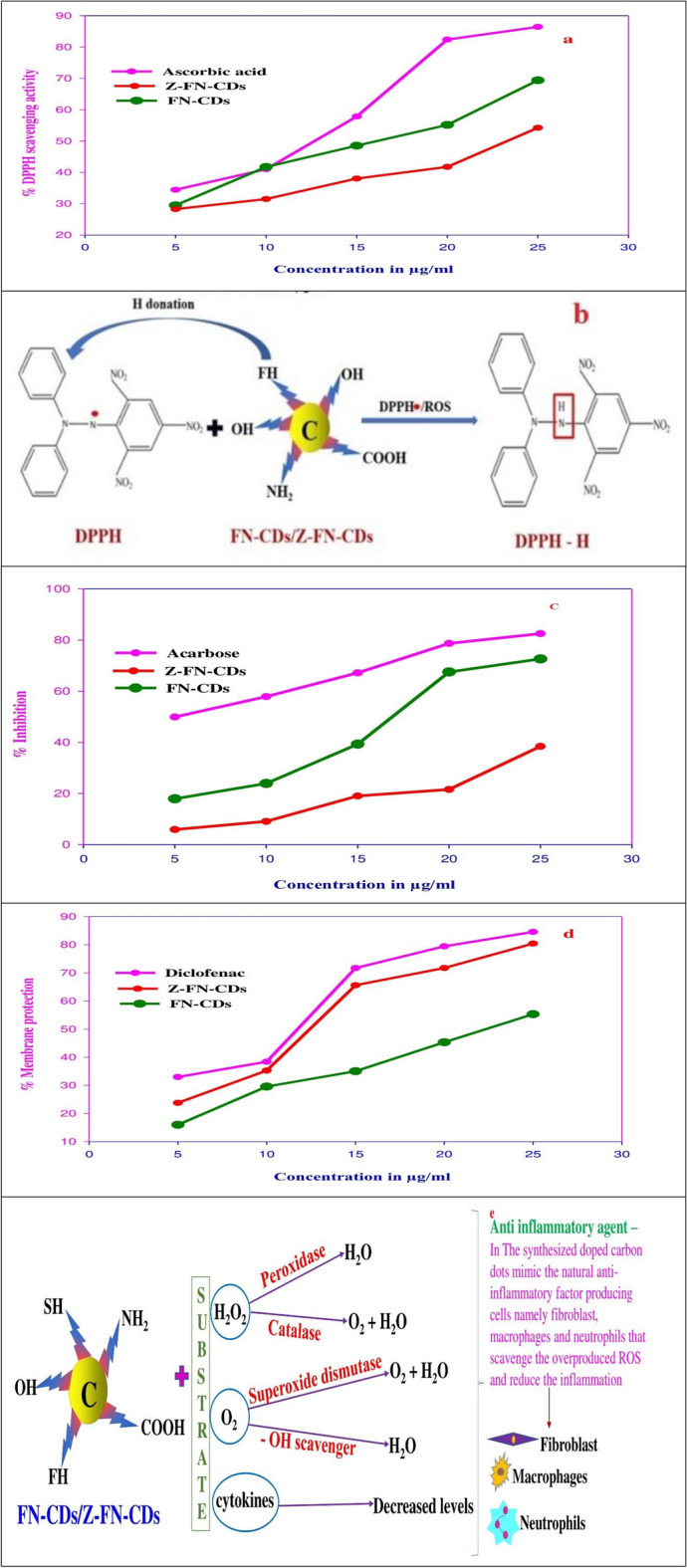
(**a**) DPPH foraging activity of FN-CDs and Z-FN-CDs in percent with ascorbic acid standard; (**b**) illustrative explanation for the mechanism of action of FN-doped carbon dots on DPPH free radical neutralization; (**c**) hypoglycemic activity of FN-CDs and Z-FN-CDs in terms of the enzyme amylase inhibition with acarbose standard; (**d**) anti-inflammatory potential in terms of human RBC (HRBC) membrane protection effected by FN-CDs and Z-FN-CDs; (**e**) mechanism of anti-inflammatory action of CDs in comparison with natural cells of human body.

Ascorbic Acid, a well-known powerful antioxidant, was used as a standard for measuring the depletion of reactive oxygen species. This anti-oxidative ability of ascorbic acid increased from 34.45 to 86.43% for the chosen concentrations, whereas the same for FN-CDs ranged between 29.49 and 69.39% and for Z-FN-CDs, it was between 28.27 and 54.24% (Fig. 7a). IC_50_ values for ascorbic acid, FN-CDs and Z-FN-CDs obtained from the DPPH-assay were 11.41 μg/mL, 15.6 μg/mL and 24.03 μg/mL, respectively (Table 1). Thus, the free radical scavenging ability of DPPH reflects its ability to accept hydrogen from the molecule of ascorbic acid and the surface functional groups of FN-CDs and Z-FN-CDs to form a stable DPPH-H complex (Fig. 7b)^51–53^. Scavenging of the DPPH-radical by FN-CDs and Z-FN-CDs in the concentration range between 5 and 25 μg/mL is inferred from the decreasing photometric absorbance values measured at 517 nm.

### Hypoglycemic activity

One of the methods used to control hyperglycemia is the inhibition of amylase. The antidiabetic potential of FN-CDs and Z-FN-CDs was determined by estimating the amylase-inhibition effect. Acarbose is a commercial amylase inhibitor and is used as a standard to compare amylase inhibition by FN-CDs and Z-FN-CDs. The percentage-inhibition-effect of FN-CDs and Z-FN-CDs was compared with that of acarbose (Fig. 7c), and the IC_50_values are tabulated in Table 1. The percentage inhibition caused by FN-CDs, Z-FN-CDs and acarbose increased from 17.94 to 72.64%, 5.9 to 38.44%, and 49.94 to 82.52%, respectively, with an increase in concentration from 50–250 μg/mL. This demonstrates that FN-CDs and Z-FN-CDs, incorporated in medications, can have considerable hypoglycemic effects.

### Anti-inflammatory activity

Experimental investigation of RBC membrane stability is ideal for evaluating anti-inflammatory properties and is easy to execute. Hypotonically-induced hemolysis of human red blood cells (HRBCs) was adopted in the study. Figure 7d depicts the effect of FN-CDs, Z-FN-CDs and standard diclofenac on the HRBC-membrane and the corresponding statistical data are presented in Table 1. The reference drug ‘diclofenac’ exhibited membrane protection to the extent of 32.9–84.5%, while the percentage of protection increased from 16 to 55% and 23 to 80% with the increase in concentration from 5 to 25 μg/mL for FN-CDs and Z-FN-CDs, respectively. The IC_50_ values for the diclofenac standard, FN-CDs, and Z-FN-CDs were 11 μg/mL, 22.2 μg/mL and 13.3 μg/mL, respectively. The anti-inflammatory effect of carbon dots derived from plants is represented in Table 2. Our results are in approximation with the findings of hemolytic analysis with MgO nanostructures^54^ and CdO–NiO composites^55^.

**Table 2:** Results of one way analysis of variance (ANOVA) for the DPPH, hypoglycemic/antidiabetic and anti-inflammatory (HRBC membrane stabilization) activity.

Assay	Standard /test	Regression equation	IC_50_ concentration (μg/ml)	Results of one way analysis of variance (ANOVA)				
				Parameter	Sum of squares (SS)	Degrees of freedom (df)	Mean square (MS)	F value
								*P* value with inference
DPPH	Ascorbic acid	y = 2.9068x + 16.826 R^2^ = 0.9528	11.41	Between groups	1175.5	2	587.7	2.0
	FN-CDs	y = 1.8644x + 20.898 R^2^ = 0.9795	15.6	Within group	3516.3	12	293.0	
	Z-FN-CDs	y = 1.2442x + 20.097 R^2^ = 0.9392	24.03	Total	4691.8	14	–	0.1 NS
Antidiabetic activity	Acarbose	y = 0.1719x + 41.459 R^2^ = 0.9834	49.73	Between groups	5870.2	2	2935.1	9.07
	FN-CDs	y = 0.306x + 1.629 R^2^ = 0.9449	158.07	Within group	3882.5	12	323.5	
	Z-FN-CDs	y = 0.1551x + 4.454 R^2^ = 0.9198	293.65	Total	9752.8	14	–	0.0 S
HRBC membrane stabilization	Diclofenac	y = 2.8832x + 18.162 R^2^ = 0.9022	11.07	Between groups	1724.0	2	862.0	1.8
	FN-CDs	y = 1.888x + 1.934 R^2^ = 0.9871	22.28	Within group	5606.0	12	467.1	
	Z-FN-CDs	y = 2.994x + 10.448 R^2^ = 0.9336	13.21	Total	7330.0	14	–	0.2 NS

### Antibacterial activity

**Figure 8:**
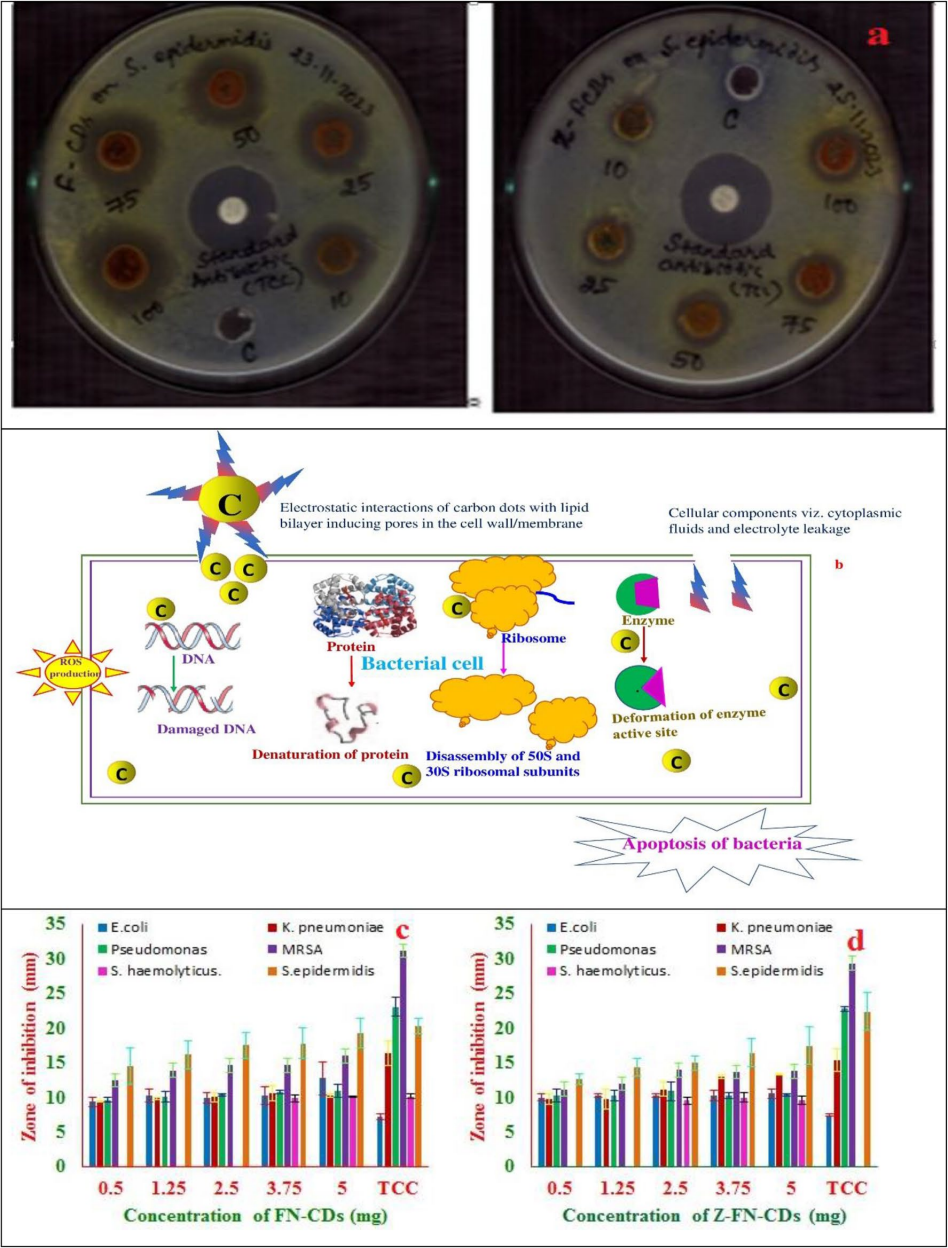
(**a**) Antibacterial activity of FN-CDs and Z-FN-CDs against *S. epidermidis*; (**b**) Schematic sketch depicting bacterial membrane/cell wall disruption by the carbon dots; (**c**) zones of Inhibition (ZOI) obtained for the tested human clinical isolates (mm) at different concentrations of FN-CDs; (**d**) zones of Inhibition (ZOI) obtained for the tested human clinical isolates (mm) at different concentrations of Z-FN-CDs.

The carbon dots were tested against three gram-positive bacteria (methicillin-resistant *S. aureus* (MRSA), *S. epidermidis*, *and S. hemolyticus)* and three gram-negative bacteria: *E. coli*, *K. pneumoniae*, *and Pseudomonas sps*. FN-CDs and Z-FN-CDs showed maximum ZOI for *S. epidermidis* (Fig. 8a, b) and MRSA. From the zones of inhibition (ZOI) obtained, it is obvious that FN-CDs and Z-FN-CDs exhibited a linear increase in antibacterial activity with an increase in the concentrations of FN-CDs and Z-FN-CDs tested on Gram + ve/ − ve bacteria (Fig. 8c and d), a reflection of their dose-dependent activity. The ZOI ranged in between 10 and 19 mm for FN-CDs (Table 3), whereas the same for Z-FN-CDs is 10 and 17 mm (Table 4). At 500 μg/well of the test substance, *S. epidermidis* was more effectively inhibited by both FN-CDs and Z-FN-CDs among the three gram-positive tested strains, followed by methicillin-resistant *S. aureus* (MRSA) and *S. hemolyticus*. Among gram-negative bacteria, *E. coli* and *K. pneumoniae* were better inhibited, followed by *Pseudomonas sps,* with the tested dose of 500 μg/well of FN-CDs (Fig. S3). Among the three studied gram-negative bacteria with Z-FN-CDs, *Pseudomonas sps.* is best inhibited, followed by *E. coli* and *K. pneumoniae.* The results of our study corroborate with the zones of clearance obtained with the carbon quantum dots^56^ prepared from *Manihot esculenta* waste peels, silver nanoparticles synthesized from *Datura metel* L.^57^, and nanocomposites of Y^3+^ and Sm^3+^ mixed metal oxides^58^ as well as ceria (Cerium IV-oxide) based nanomaterials^59^.

**Table 3:** Results of one way analysis of variance (ANOVA) for the antibacterial activity of FN-CDs.

Name of the pathogen	ZOI in mm expressed as mean ± SD of triplicates (FN-CDs)							F value
	Std. Ab	1	2	3	4	5	6	*P* value with inference
*E. coli*	7.2 ± 0.4	9.3 ± 0.6	10.3 ± 0.8	9.9 ± 0.8	10.2 ± 1.2	12.8 ± 2.3	0 ± 0	4.8
								0 **S**
*K. pneumonia*	16.4 ± 1.8	9.3 ± 0.1	9.9 ± 0.1	10.1 ± 0.7	10.7 ± 1.1	10.3 ± 0.2	0 ± 0	3.09
								0.03 **S**
*Pseudomonas sp.*	23.1 ± 1.4	9.6 ± 0.4	10.1 ± 0.8	10.3 ± 0.2	10.7 ± 0.2	11.0 ± 0.9	0 ± 0	3.85
								0.01 **S**
*S. aureus*	31.2 ± 0.7	12.4 ± 0.5	13.8 ± 0.1	14.6 ± 0.9	14.6 ± 0.4	15.9 ± 1.1	0 ± 0	15.02
								0 **S**
*S. hemolyticus*	0 ± 0	0 ± 0	0 ± 0	9.9 ± 0.4	10.1 ± 0.1	10.1 ± 0.3	0 ± 0	2272.6
								0 **S**
*S. epidermidis*	20.3 ± 1.1	14.5 ± 2.5	16.1 ± 2.0	17.5 ± 1.8	17.8 ± 2.2	19.3 ± 2.2	0 ± 0	3.3
								0.02 **S**

Ab: Antibiotic; The *p* value is significant at *p* < 0.05; ** denotes *p* < 0.01; * denotes *p* < 0.05; S—significant; NS—Non-significant.

**Table 4:** Results of one way analysis of variance (ANOVA) for the antibacterial activity of Z-FN-CDs.

Name of the pathogen	ZOI in mm expressed as mean ± SD of triplicates (Z-FN-CDs)							F value
	Std. Ab	1	2	3	4	5	6	*P* value with inference
*E. coli*	7.4 ± 0.1	10.0 ± 0.4	10.2 ± 0.2	10.3 ± 0.2	10.2 ± 0.7	10.6 ± 0.6	0 ± 0	0.9
								0.4 **NS**
*K. pneumonia*	15.4 ± 1.5	9.7 ± 0.7	9.7 ± 1.5	11.1 ± 1.0	12.9 ± 0.3	13.3 ± 0.1	0 ± 0	13.7
								0 **S**
*Pseudomonas sp.*	22.8 ± 0.4	10.3 ± 0.9	10.3 ± 0.6	10.9 ± 1.3	10.3 ± 0.5	10.4 ± 0.1	0 ± 0	0.4
								0.7 **NS**
MRSA	29.4 ± 1.8	11.1 ± 0.7	12.0 ± 0.8	13.9 ± 0.8	13.6 ± 0.4	13.7 ± 0.5	0 ± 0	15.02
								0 **S**
*S. hemolyticus*	0 ± 0	0 ± 0	0 ± 0	9.6 ± 0.4	9.9 ± 0.6	9.5 ± 0.5	0 ± 0	695.5
								0 **S**
*S. epidermidis*	22.4 ± 2.7	12.7 ± 0.7	14.3 ± 1.2	15.0 ± 0.9	16.5 ± 2.0	17.4 ± 2.6	0 ± 0	5.7
								0 **S**

Ab: Antibiotic; The *p* value is significant at *p* < 0.05; ** denotes *p* < 0.01; * denotes *p* < 0.05; S—significant; NS—Non-significant.

### Cell viability

**Figure 9:**
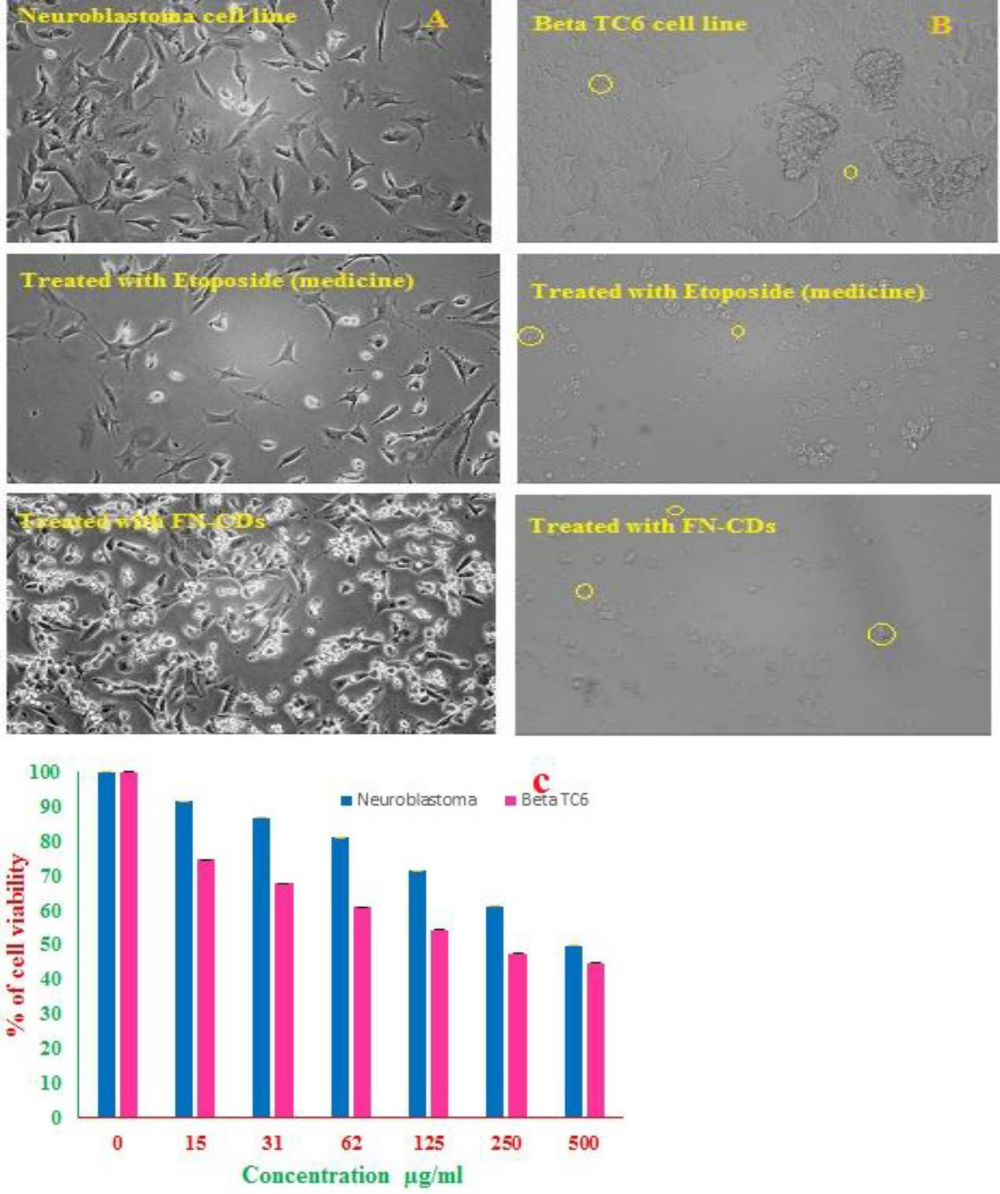
(**a**) Phase contrast imaging of neuroblastoma cells; (**b**) beta TC6 cell lines in presence of FN-CDs; (In vitro cell line study); (**c**) cell viability percent of neuroblastoma and beta TC6 cell lines on exposure to different concentrations of FN-CDs.

Neuroblastoma and beta-TC6-cell lines were treated with different concentrations (15–500 μg/mL) of FN-CDs, and cell viability was measured by the MTT-assay after 24 h of incubation (Fig. 9a). The different concentrations of FN-CDs (15, 31, 62, 125, 250 and 500 μg/mL) showed a decrease in the percent-cell-viability in the order of 91.68, 86.67, 80.98, 71.39, 61.29 and 49.74 respectively for neuroblastoma-cells, with reference to the untreated control-cells. The same concentrations of FN-CDs for beta TC6-cells showed a similar trend with viability values of 74.65, 67.7, 60.96, 54.3, 47.5 and 44.7, respectively, in comparison to the untreated control cells (Fig. 9b). Of the two cell lines tested, the neuroblastoma-cells demonstrated more than 80% viability only up to 62 μg/mL concentration of FN-CDs (Fig. 9c), and beyond this, the viability dropped steeply. This trend could be positively manipulated for effecting the desired therapeutic function of either proliferation for tissue regeneration and/or inducing cell death, as required in malignancy, just by varying the concentrations.

## Discussion

Carbon dots were prepared by solvothermal process using *Moringa oleifera L*., *Chromolaena odorata L*., *Tridax procumbens L*., *Tinospora cordifolia L*. and *Lantana camara L*. leaf powders*.* The leaves containing cellulose, proteins, phenols, and falvonoids act as natural and remarkable green precursors for the carbon dots’ production. At 180 °C, the cellulose is hydrolyzed, phenols are degraded, and proteins and flavonoids are destabilized. DFBD is used as a precursor for the synthesis of carbon dots because of the high stability of fluorine in aromatic rings and the characteristics that the amino groups impart to the N-bearing units under solvothermal conditions, including positive charge localization, Schiff-base-Fragment, and N-heterocycle. The presence of C–F- and C–N-Bonds in the structure of carbon dots suggests the presence of F and N. The organic components in plant-leaf-extract provide the carboxyl- and hydroxyl-functional groups seen in the carbon-dots.

The X-ray diffractogram of FN-CDs revealed a crystalline structure with strong pulse amplitudes (PAs) at 2*θ*-values of 15.4°, 27.56°, and 31.6°. Z-FN-CDs showed less pronounced PAs at 19.1° and 42.5° (JCPDS 26-1076). XRD data revealed lattice constant, strain, and dislocation density for both FN-CDs and Z-FN-CDs. Low strain and dislocation densities of FN-CDs indicate their potential biomedical applications. An ideal zeta potential value for biomaterial-derived carbon dots correlates with enhanced biological functions like wound healing and tissue regeneration. FN-CDs showed a sharp peak at 19.4 mV, while Z-FN-CDs showed maximum peak intensity at 0.3 mV.

DPPH is a reliable, simple, and well-established method for estimating antioxidant activity. The free-radical-scavenging-ability prevents damage caused by the generation of free-radicals and maintains proper cellular function. In the presence of antioxidants, the purple-colored DPPH-Solution changes to the yellow-colored DPPH-H-Solution. The capacity of an antioxidant is deduced from its ability to donate hydrogen-ions^51–53^. Enzymatic antioxidants, exemplified by peroxidase, ascorbate peroxidase, and catalase, remove reactive species by transforming the oxidation products to water. These antioxidants are released from cells cultivated in a medium containing the cofactors copper, zinc, and manganese. The second class of non-enzymatic antioxidants includes vitamins, polyphenols, phenolic acids, flavonoids, ascorbic acid and glutathione; these compounds stop oxidation by inhibiting ROS-chain reactions^60^. DPPH (2,2-diphenyl-1-picrylhydrazyl) is a persistent free radical with a purple hue, and its assay is a quick, simple, and affordable way to assess the radical scavenging activity of non-enzymatic antioxidants. Carbon dots synthesized from plants are categorized as non-enzymatic radical scavengers^61^.

Carbon dots scavenge reactive species of nitrogen and hydroxyls (·OH), which otherwise impair cellular processes^62^. The surface of carbon dots releases hydrogen, which is then picked up by the DPPH-free radical, which has core nitrogen and two pairs of non-bonding electrons embraced by three benzene rings. While the unpaired electrons on the CDs are shifted through chemical bond rearrangement or resonance in aromatic domains, the presence of amino (–NH_2_, –NH–), carboxyl (–COOH), and hydroxyl (–OH) groups that supply hydrogen facilitates this reduction pathway of DPPH· to DPPH-H. ·OH-Radicals harm biomolecules including lipids, proteins, and nucleic acids through oxidative damage, and they are the prime cause of stress in biological units. Carbon dots can participate in electron transfer reactions with these ·OH-radicals and eliminate them by transformation into less reactive molecules through electron transfer^61^. The effectiveness of carbon dots in neutralizing many ·OH-radicals is reportedly increased by redox-recycling^63,64^. Furthermore, singlet-oxygen-molecules (ROS-radicals), which are responsible for intense oxidative damage, are neutralized by interaction with the excited states of carbon dots^63,64^. The antioxidant capabilities of C-dots are determined from their excited-state quenching^62,65^.

Amylase breaks down the starch into maltose (disaccharide) and glucose (monosaccharide) units. *Zingiberis carbonisata-*based carbon dots can reduce blood-glucose-levels in diabetic mice. In addition, these dots decrease the levels of inflammatory cytokines and suppress protein expression^66^. Carbon dots made from *Artemisiae Argyi Folium* (AAF) carbonisata have anti-inflammatory properties because they inhibit the expression of inflammatory mediators and lower blood-glucose levels in mice^67^.

Anti-inflammatory drugs used to combat inflammation stabilize the lysosome membrane and prevent the release of acidic lysosomal enzymes in the cytosol. The bioactive compounds and functional groups present on carbon dots are presumed to inhibit the lysis of the RBC membrane. The FN-CD-loaded HRBCs are protected from being lysed by the induced hypotonic environment, indicating that the membranes of the RBCs are stabilized by the doped-carbon-dots. The carbon dots derived from plants exhibit an anti-inflammatory effect due to their ability to forage reactive radicals (Fig. 7e) and interact with components of signaling pathways for inflammation by down regulating the pro-inflammatory mediators like “TNF-α, IL-1α, IL-1β, IL-2, IL-6, IL-8, IL-12, and IFN-γ receptors” and empower the immune system^68–70^. The carbon dots synthesized from broccoli using the hydrothermal method were tested in zebrafish for their anti-inflammatory activity and were found to reduce the expression of “TNF-α” and “IL-6”. The expressions of glutathione peroxidase (GPX-4) and superoxide dismutase (SOD) are upregulated, thus increasing the antioxidant activity and reducing inflammation^7^. The carbon dots derived from mulberry silkworm cocoon carbonisata (MSCC) showed anti-inflammatory activity in a lipopolysaccharide-induced inflammation model, which closely resembles sepsis in humans^71^. Cerium-doped carbon nanodots are used as anti-inflammatory agents in mice for wound healing^72^.

The amines and amides present on the surface of carbon dots were inferred to affect the antimicrobial activity of dots because of the electrostatic interactions between the protonated groups and lipids present in the bacterial cell membrane. Furthermore, the F^−^ and NH_2_^+^ groups on the surfaces of doped CDs react with the cell wall/cell membrane components of the bacteria and affect their lysis. The rise in multidrug-resistant (MDR) bacterial wound-infections to available antibiotics, accompanied by a continuous decline in antibiotic development, is a major global issue. Therefore, there is a need to synthesize the agents that act against the MDR-bacterial strains. The first step of interaction between the antimicrobial agents, FN-CDs and Z-FN-CDs in this study, and bacteria is direct, through electrostatic action and/or chemical conjugations. The study results are in corroboration with the two proposed mechanisms that exist for CDs to attach to the bacterial cell membrane and cause physical or mechanical damage, viz. the rupture of the bacterial cell wall, allowing the CDs to enter the internal membranes, the membrane collapse due to loss of cytoplasmic fluids and electrolytes^73^, and the electrostatic interactions for surface adherence^74^. The amine groups present on CDs denatures the DNA, resulting in apoptosis of the cell^75^. Xiang et al.^76^ reported 99.9% inactivation of *E. coli* and *S. aureus* in wounds applied with dressings containing cabon-ZnO hybrid nanoparticles added to the folic acid-conjugated polydopamine hydrogel.

**Figure 10:**
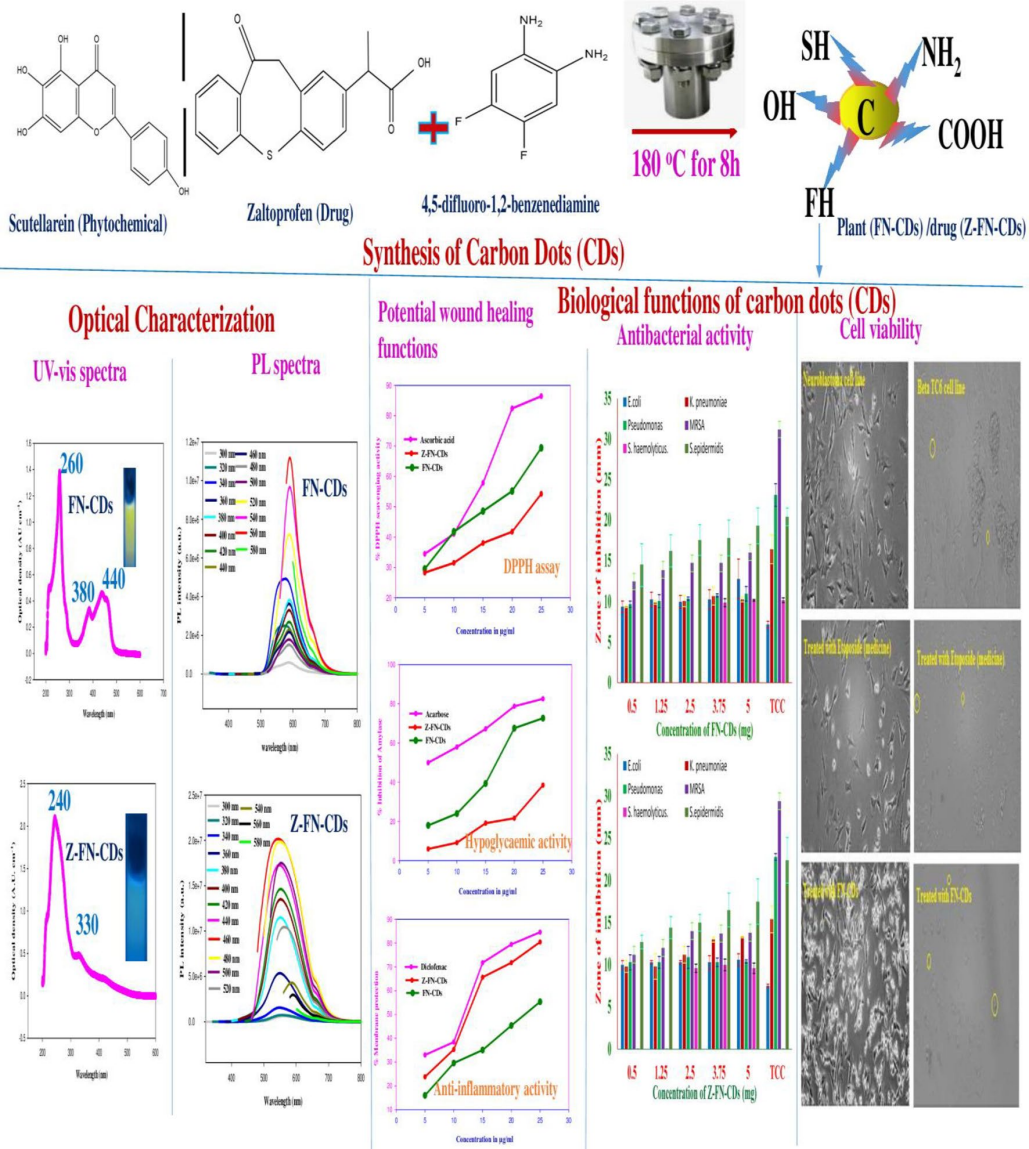
Illustration summarizing the synthesis, characterization and biological functions of the synthesized heteroatom doped carbon dots.

Though mainly a defensive mechanism, inflammation can sometimes promote the growth, invasion, and metastasis of tumour-cells. Under these physiological conditions, carbon dots could be used as therapeutics at a certain concentration and beyond for inducing apoptosis. Below this threshold value, CDs could be deployed as antioxidant and anti-inflammatory agents and harness their potential for lipopolysaccharide-induced inflammation, cartilage/bone tissue regeneration, and wound healing^46^. The toxicity and fluorescence of carbon dots are significantly influenced by their size^77^. In a nut-shell, the significance of the current study depicted in Fig. 10 can be deduced from the reported literature summarized (Table 5) from the investigative work of a few other authors.

**Table 5:** Summarized findings of a few chosen research articles highlighting the importance of plant derived carbon dots and a commercial drug (Levofloxacin) in biomedical applications.

S. no	Type of material	Salient features corroborating with the current study	References
1	Carbon quantum dots from *Manihot esculenta* waste peels	Optical properties and antibacterial activity	^56^
2	Carbon quantum dots derived from sugarcane industrial wastes	Nonlinear optical (NLO) devices, bioimaging, and pharmaceutical applications	^16^
3	Carbon quantum dots extracted from natural honey	Optical and antibacterial properties	^17^
4	Blocks decorated with garlic peel biochar nanoparticles	Degradation of methyl orange and its antioxidant activity	^53^
5	Carbon dots from levofloxacin hydrochloride	Optical properties and antibacterial potential	^75^
6	Carbon dots from *Azadirachta indica*	Antimicrobial, antioxidant, bioimaging	^3^
7	FN-CDs synthesized from plants and Z-FN-CDs from zaltoprofen drug	Optical, antioxidant, anti-inflammatory, antibacterial, hypoglycemic, and biocompatibility properties	Present study

## Conclusions

Heteroatom-doped carbon dots were synthesized from the vegetative parts of medicinal plants viz. *Moringa oleifera L./Chromolaena odorata L./Tridax procumbens* L./*Tinospora cordifolia L.* and *Lantana camara* L., with the precursor DFBD via a solvothermal reaction. Simultaneously, carbon dots of the commercial anti-inflammatory drug “zaltoprofen” were synthesized using the same process and the precursor. The FN-CDs and Z-FN-CDs showed an excitation-wavelength (300–580 nm) dependent emission-wavelength ranging from 550 to 600 nm. The optimal/maximum emission peak for FN-CDs was observed at 600 nm for an excitation wavelength of 560 nm, whereas the same peak for Z-FN-CDs was observed at 550 nm against an excitation wavelength of 460 nm. FN-CDs showed excellent antioxidant, anti-inflammatory, hypoglycemic, and antibacterial properties. Higher antioxidant (69.4%, 54.2%) and anti-inflammatory potentials (55.3%, 80.4%) at 25 μg/mL concentrations were obtained for FN-CDs and Z-FN-CDs, respectively. Antibacterial activity of FN-CDs against three-gram +ve *S. aureus* (MRSA), *S. epidermidis*, *and S. hemolyticus* and three-gram-ve *E. coli*, *K. pneumoniae*, *and Pseudomonas sps*. demonstrated mean zones of inhibition in millimeters of 15.9 ± 1.1, 19.3 ± 2.2, 10.1 ± 0.3, 12.8 ± 2.3, 10.3 ± 0.2 and 11 ± 0.9, respectively. The antibacterial activity of Z-FN-CDs against three gram-positive *S. aureus* (MRSA), *S. epidermidis*, *and S. hemolyticus* and three gram-negative *E. coli*, *K. pneumoniae*, *and Pseudomonas sps* showed mean zones of inhibition in millimeters of 13.7 ± 0.5, 17.4 ± 2.6, 9.5 ± 0.5, 10.6 ± 0.6, 13.1 ± 0.1 and 10.4 ± 0.1 respectively. Cell viability, measured by the MTT Assay, revealed that concentrations higher than 500 μg/mL of carbon dots killed the cells, suggestive of their anti-cancer property.

### Futuristic perspective

Carbon dots (CDs), with their optoelectronic properties and tunable fluorescence, offer exceptional biomedical applications such as bioimaging, biosensing, and regenerative medicine. These materials exhibit controlled architecture, low toxicity, biodegradability, and therapeutic properties especially when derived from medicinal plants, making them ideal for drug delivery in anti-inflammatory, antibacterial, anticancer and wound healing treatments. Further, their biocompatibility and dual-emission fluorescence make them ideal for accurate diagnostics, targeted imaging and mitigating drug-related side effects. Their green synthesis is ecofriendly, cost-effective and in toto, CDs represent a revolutionary nanomaterial with immense potential in future biomedical technologies.

## Methods

Leaves of the plants *Lantana camara* L., *Tinospora cordifolia* L., *Tridax procumbens* L. and *Moringa oleifera* L. were collected from within the premises of RVR and JC College of Engineering, Chowdavaram, Guntur, Andhra Pradesh, India, while the leaves of *Chromolaena odorata* L. were collected from Turakalakota, a small village in the Srikakulam district of Andhra Pradesh, India. The taxonomic status of the chosen plants for the study was authenticated by the Head, Department of Botany, Bapatla College of Arts and Sciences, Bapatla, Andhra Pradesh, India. Further, the experimental research and the collection of plant material (Leaves) are in compliance with the institutional and national guidelines of India. The collected leaves of the mentioned plants were rinsed with tap water to remove dirt, shaded and ground in a blender.

The experimental protocols adopted in the study are standard procedures and were approved by the institutional ethics committee (IEC).

All chemical reagents including solvents used in the study were of analytical grade with 99.9% purity. 4, 5-Difluoro-1, 2-benzenediamine (DFBD), (DPPH), (3-(4,5-Dimethylthiazol-2-yl)-2,5-Diphenyltetrazolium Bromide (MTT), ascorbic acid, ethyl and methyl alcohols, dinitro salicylic acid (DNS), phosphate buffers are some of the major chemical reagents employed in the study. Double distilled water was used for the preparation of solutions, wherever necessary.

### Doped carbon dots production using plant extracts (FN-CDs) and zaltoprofen (Z-FN-CDs)

The precursor, 4, 5-Difluoro-1, 2-benzenediamine (DFBD), which weighed 480 mg, was dissolved in 15 mL of ethyl alcohol. A separate 1.5 g of plant powder (including *Lantana camara* L., *Tinospora cordifolia* L., *Tridax procumbens* L., *Moringa oleifera* L. and *Chromolaena odorata* L.) was dissolved in 45 mL of ethyl alcohol and agitated for 20 min. Plant extract and DFBD were then combined in equal amounts (15 mL of each) and placed in a stainless-steel-autoclave lined with Teflon. After eight hours of heating at 180 °C, the mixture was cooled. Insoluble particles were removed by filtration across the nylon membrane (0.2 μm pore diameter), followed by examination under UV-Lamp. Separately, 1.5 g zaltoprofen and 480 mg DFBD were solubilized in 15 mL of ethyl alcohol each. After that, the two solutions were combined, put in a stainless-steel-autoclave with a Teflon-lining, and treated as directed for doped-CDs^32^.

### Characterization of the carbon-dots

The synthesized carbon dots were observed under a UV-Lamp (Analytik Jena, CA, USA) in Gel-Doc-Equipment and noted their fluorescence color. The carbon dots were analytically characterized by UV–vis (UV2000U, Labindia), fluorescence (Horiba, JobinVyon), Fourier transform infrared (FTIR; Shimadzu, Miracle 10), and X-ray diffraction (Rigaku diffractometer Ultima IV 2036E202) methods. The zeta potential was evaluated using a particle-size-analyzer (Horiba, SZ-100). While the shape and form of the carbon dots was observed by high resolution transmission microscopy (HRTEM, 200 kV), Image J and Match 4 soft wares were used to calculate the diameter, lattice constant, strain, dislocation density and d-spacing of the carbon dots. Cell survival was tested by MTT-Assay and estimated with the aid of a microplate reader (Bio-Rad, USA).

Carbon Dots’ DPPH-radical forage activity was assessed through the application of a slightly altered version of Hsu et al.^78^. 5 mL of DI-Water, 3 mL of 0. 3 mM DPPH in methyl-alcohol, and the designated doses of FN-CDs and Z-FN-CDs in micrograms per milliliter of 5, 10, 15, 20, and 25 were added to experimental tubes. These were then gently vortexed and left in an aphotic environment at ambient temperature for half an hour. In parallel with the experimental tubes, a control was set up with the exclusion of test material. As positive controls, various aliquots of ascorbic acid in the concentrations corroborating with the test samples were employed for the conduct of the assay. The Reduction of DPPH was detected by a visible photometer at 517 nm (Labindia, Model: UV3200).

1 $$ {\text{Free}}\;{\text{radical}}\;{\text{scavenging}}\;{\text{in}}\;{\text{percent}}\;{\text{of}}\;{\text{DPPH}} = \left( {Ac - At} \right)/Ac \times 100 $$

The absorbance measurements of the control and test/standard are A_c_ and A_t_ respectively. The concentration of FN-CDs/Z-FN-CDs (μg/mL) was plotted against the percentage of DPPH-scavenging-activity on the graph. From the regression equation, the IC_50_ was calculated and ANOVA was performed.

### Hypoglycemic activity

The “amylase inhibition” was estimated using the method proposed by Bernfeld, with minor changes^79^. Different concentrations in micrograms per milliliter of FN-CDs/Z-FN-CDs, viz. 50–250, were added to 1 mL of buffer containing KH_2_PO_4_ and NaOH (pH 11.0), 1 mL of 1% aqueous starch, and 1 mL of human salivary amylase and incubated at 20 °C for 5 min. After adding 2 mL of di-nitro salicylic acid (DNS) reagent to every experimental tube, the tubes were placed at 70 °C in a hot-water-bath for 10 min, followed by cooling with flowing tap-water. Lastly, 10 mL of water were mixed with the brown reduction product that had been collected in the experimental tubes, and an optical spectrophotometer was used to measure the OD-values at 540 nm. Everything was prepared for a blank, with the exception of the amylase-enzyme. A control was placed without the test sample (carbon dots), which represents 100% enzyme activity. A commercial antidiabetic drug, acarbose (50, 100, 150, 200, and 250 μg), was used as a positive control. One milliliter of starch (1%) dissolved in phosphate buffer (0.02 M, pH 6.9) and one milliliter of human salivary amylase were added to the acarbose solution, and it was then incubated at 20 °C for five minutes. After adding 2 mL of DNS-reagent, the previously described procedures for the doped CDs were then carried out. The percent inhibition of amylase by CDs and acarbose is expressed as follows:

2 $$ \% \;{\text{Amylase}}\;{\text{Inhibition}} = \left( {{\text{A}}_{{\text{c}}} - {\text{A}}_{{\text{t}}} } \right)/{\text{A}}_{{\text{c}}} \times {1}00 $$

The optical density values of the test and the control samples are denoted by Ac and At, respectively.

### Membrane stability

In vitro testing of Carbon dots’ anti-inflammatory properties was done using stabilization of Human Red Blood Cell membranes^80^. Heparinized, purple-capped ethylene diamine tetra acetic acid (EDTA) tubes were used to hold the 2 mL of blood that were taken from each of the healthy volunteers, who had not taken any medicine in the previous two weeks, with their informed agreement. After centrifuging the test sample for 15 min at 3000 revolutions per minute to separate the RBC and plasma, the recovered RBCs were repeatedly washed with an isotonic solution of NaCl (0.9%) and then centrifuged again. Using the same isotonic NaCl solution, the collected centrifuge was diluted to a 10% solution (v/v). Informed consent was obtained from all human subjects who participated as volunteers in the study, as per institutional ethics committee (IEC) guidelines.

### Induced hemolysis

To various concentrations of FN-CDs/Z-FN-CDs (5–25 μg/mL), 0.5 mL of human RBC (HRBC) suspension, 2 mL of 0.2% NaCl (hyposaline) and 2 mL of 0.15 mM phosphate buffer at pH 7.4 were added. For the control, deionized (DI) water was added instead of hypotonic NaCl. Diclofenac was used as the standard reference. The Analysis tubes were maintained at 37 °C for 30 min and centrifuged at 3000 rpm for 15 min. Hemoglobin content in the supernatant was estimated spectrophotometrically (λ (nm) = 560). The percentage hemolysis was determined from the expression:

3 $$ {\text{Hemolysis}}\% = \left[ {\left( {{\text{Absorbance}}\;{\text{of}}\;{\text{sample}}} \right)/\left( {{\text{Absorbance}}\;{\text{of}}\;{\text{control}}} \right)} \right] \times 100 $$

4 $$ {\text{Percentage}}\;{\text{of}}\;{\text{protection}} = 100\; -{\text{hemolysis}}\;{\text{in}}\;{\text{percentage}} $$

### Antibacterial-activity

The antibacterial capacity of FN-CDs/Z-FN-CDs was tested against pathogenic human clinical isolates that included methicillin-resistant *Staphylococcus aureus* (MRSA), *Staphylococcus epidermidis*, and *Staphylococcus hemolyticus under gram positive, while Escherichia coli*, *Klebsiella pneumoniae*, *Pseudomonas sps* were under gram negative. They were procured from the microbiology department of the local multispecialty hospital and medical college, following the institutional ethical committee guidelines. Nutrient-agar-medium was used as the cultivation medium for the bacterial strains. After 2 passes, 0.6 OD bacterial cultures were used for antibacterial activity evaluation. The clinical isolates were cultured in peptone water (Hi-Media) medium under aerobic conditions at 37 °C for 24 h. With the help of a sterile corkborer, six wells of 6 mm depth were punched. Then, FN-CDs/Z-FN-CDs in DMSO (50 mg/mL) were taken in volumes of 10, 25, 50, 75, and 100 μl, and added in a clockwise direction in the order of wells labeled from 1 to 5. A 50 μl negative control consisting of dimethyl sulfoxide was added in the sixth well. A 75/10 mcg ticarcillin/clavulanic acid (TCC) antibiotic disk was placed in the center of the plate, incubated at 37 °C overnight and recorded the zones of clearance.

### Biocompatibility testing with cell lines

Neuroblastoma and beta-TC6-cells were acquired from the National Centre for Cell Science (NCCS), Pune, India. Hams’ F12K-medium for neuroblastoma-cells, DMEM (Dulbecco’s modified Eagle-medium) and F-12 (Hams’ F-12 nutrient mixture) in 1:1 proportion for beta-TC6-cells, were added with 10% FBS, and antibiotics of the classes aminoglycoside (streptomycin) and β-lactam (penicillin). A final concentration of 1 × from a 100% stock of either of the media was used in strict aseptic conditions throughout the experimentation. The cells were treated with Trypsin–EDTA after the attainment of fluent growth. Then 10^6^ cells of each of the chosen cell lines were seeded in standard culture plates of 96 wells and kept in an incubator with atmospheric conditions of 95% humidity and 5% carbon dioxide. The biocompatibility test was performed in triplicate. Different concentrations of FN-CDs, namely 15 μg, 31 μg, 62 μg, 125 μg, 250 μg, and 500 μg, were applied to a 100 μl volume of cells and incubated with 50 μl of (3-(4,5-Dimethylthiazol-2-yl)-2,5-Diphenyltetrazolium Bromide (MTT) for three hours at 37 °C. After incubation, to each test tube phosphate-buffer saline of 200 μl was added, and any residual MTT, if found, was carefully removed. For solubilization, 200 μl of acid-propanol were then added and left in the dark for the entire night. A phase-contrast-microscope was used to view the cells, while a microplate reader read the absorbance at 570 nm. After 24 h, the absorbance of the control-cells (those not receiving treatment) was fixed at 100% Viability, and the percentage of vital-cells in the other treatment-groups was determined using the formula.

5 $$ {\text{Percent}}\;{\text{Viability}} = \left[ {\left( {{\text{OD}}\;{\text{of}}\;{\text{Control}}{-}{\text{OD}}\;{\text{of}}\;{\text{sample}}} \right)} \right]/\left[ {{\text{OD}}\;{\text{of}}\;{\text{Control}}} \right] \times 100 $$

### Supplementary Information

Supplementary Information 1.

Supplementary Information 2.

## Data Availability

The raw data of the current study are compiled and provided as a supplementary file.
