# Supplementary material for: Heteroatom-doped carbon dots from medicinal plants as novel biomaterials for as-use biomedical applications in comparison with synthetic drug, zaltoprofen
Source: Sci Rep. 2024 Jun 7;14:13160. doi: 10.1038/s41598-024-63700-w (PMC11161473; doi:10.1038/s41598-024-63700-w)
Supplement: Supplementary file 1 — Supplementary Information 1. [file 41598_2024_63700_MOESM1_ESM.zip › Raw data of scientific reports/Fig. 6a DPPH activity.docx]

| FN- CDs - DPPH | O.D. (1) | O.D. (2) | O.D.  (3) | Average | % |
| --- | --- | --- | --- | --- | --- |
| 5 | 0.96 | 0.947 | 0.851 | 0.91 | 29.49 |
| 10 | 0.785 | 0.768 | 0.726 | 0.75 | 41.74 |
| 15 | 0.667 | 0.65 | 0.671 | 0.66 | 48.54 |
| 20 | 0.593 | 0.61 | 0.55 | 0.58 | 55.16 |
| 25 | 0.267 | 0.49 | 0.44 | 0.39 | 69.39 |
|  |  |  |  |  |  |
|  |  |  |  |  |  |
|  |  |  |  |  |  |
|  |  |  |  |  |  |
| **Z - FN -CDs DPPH** | O.D. (1) | O.D. (2) | O.D.  (3) | Average | % |
| 5 | 0.72 | 0.71 | 0.713 | 0.71 | 28.27 |
| 10 | 0.671 | 0.68 | 0.696 | 0.68 | 31.49 |
| 15 | 0.635 | 0.659 | 0.617 | 0.63 | 38.04 |
| 20 | 0.587 | 0.592 | 0.561 | 0.58 | 41.76 |
| 25 | 0.451 | 0.467 | 0.449 | 0.45 | 54.24 |
|  |  |  |  |  |  |
|  |  |  |  |  |  |
|  |  |  |  |  |  |
| **Ascorbic acid - DPPH** | O.D. (1) | O.D. (2) | O.D.  (3) | Average | % |
| 5 | 0.748 | 0.66 | 0.636 | 0.68 | 34.45 |
| 10 | 0.605 | 0.57 | 0.657 | 0.61 | 41.02 |
| 15 | 0.467 | 0.394 | 0.452 | 0.43 | 57.84 |
| 20 | 0.125 | 0.119 | 0.296 | 0.18 | 82.4 |
| 25 | 0.11 | 0.09 | 0.217 | 0.13 | 86.43 |

Fig. 6a DPPH activity
