# Supplementary material for: Heteroatom-doped carbon dots from medicinal plants as novel biomaterials for as-use biomedical applications in comparison with synthetic drug, zaltoprofen
Source: Sci Rep. 2024 Jun 7;14:13160. doi: 10.1038/s41598-024-63700-w (PMC11161473; doi:10.1038/s41598-024-63700-w)
Supplement: Supplementary file 1 — Supplementary Information 1. [file 41598_2024_63700_MOESM1_ESM.zip › Raw data of scientific reports/Fig. 6c Hypoglycemic activity.docx]

Fig. 6c Hypoglycemic activity

| **FN-CDs Hypoglycemic activity** | O.D. (1) | O.D. (2) | O.D.  (3) | Average | % |
| --- | --- | --- | --- | --- | --- |
| 50 | 0.96 | 0.94 | 0.99 | 0.96 | 17.94 |
| 100 | 0.89 | 0.85 | 0.93 | 0.89 | 23.93 |
| 150 | 0.71 | 0.74 | 0.68 | 0.71 | 39.31 |
| 200 | 0.38 | 0.41 | 0.35 | 0.38 | 67.52 |
| 250 | 0.29 | 0.34 | 0.33 | 0.32 | 72.64 |
|  |  |  |  |  |  |
|  |  |  |  |  |  |
|  |  |  |  |  |  |
|  |  |  |  |  |  |
| **Z-FN-CDs Hypoglycemic activity** | O.D. (1) | O.D. (2) | O.D.  (3) | Average | % |
| 50 | 1.026 | 1.147 | 1.176 | 1.116 | 5.9 |
| 100 | 0.954 | 1.121 | 1.161 | 1.078 | 9.1 |
| 150 | 0.93 | 1.014 | 0.96 | 0.96 | 19.05 |
| 200 | 89 | 0.96 | 0.94 | 0.93 | 21.58 |
| 250 | 0.74 | 0.76 | 0.71 | 0.73 | 38.44 |
|  |  |  |  |  |  |
|  |  |  |  |  |  |
|  |  |  |  |  |  |
| Acarbose | O.D. (1) | O.D. (2) | O.D.  (3) | Average | % |
| 50 | 0.416 | 0.438 | 0.409 | 0.421 | 49.94 |
| 100 | 0.353 | 0.342 | 0.368 | 0.354 | 57.9 |
| 150 | 0.284 | 0.275 | 0.271 | 0.276 | 67.18 |
| 200 | 0.192 | 0.175 | 0.172 | 0.179 | 78.71 |
| 250 | 0.151 | 0.147 | 0.143 | 0.147 | 82.52 |
