# Supplementary material for: Heteroatom-doped carbon dots from medicinal plants as novel biomaterials for as-use biomedical applications in comparison with synthetic drug, zaltoprofen
Source: Sci Rep. 2024 Jun 7;14:13160. doi: 10.1038/s41598-024-63700-w (PMC11161473; doi:10.1038/s41598-024-63700-w)
Supplement: Supplementary file 1 — Supplementary Information 1. [file 41598_2024_63700_MOESM1_ESM.zip › Raw data of scientific reports/Fig. 6d Antiinflammatory activity.docx]

| **FN - CDs - Anti inflammatory** | O.D. (1) | O.D. (2) | O.D.  (3) | Average | % |
| --- | --- | --- | --- | --- | --- |
| 5 | 0.84 | 0.79 | 0.89 | 0.84 | 16 |
| 10 | 0.8 | 0.91 | 0.74 | 0.81 | 29.57 |
| 15 | 0.79 | 0.72 | 0.85 | 0.78 | 35.05 |
| 20 | 0.76 | 0.68 | 0.84 | 0.76 | 45.33 |
| 25 | 0.63 | 0.57 | 0.69 | 0.63 | 55.32 |
|  |  |  |  |  |  |
|  |  |  |  |  |  |
| **Z-FN-CDs Anti inflammatory** | O.D. (1) | O.D. (2) | O.D.  (3) | Average | % |
| 5 | 1.3 | 1.1 | 1.5 | 1.3 | 23.8 |
| 10 | 1.01 | 0.96 | 1.07 | 1.01 | 35.26 |
| 15 | 0.54 | 0.59 | 0.48 | 0.53 | 65.59 |
| 20 | 0.44 | 0.49 | 0.38 | 0.43 | 71.72 |
| 25 | 0.28 | 0.24 | 0.32 | 0.28 | 80.42 |
|  |  |  |  |  |  |
|  |  |  |  |  |  |
|  |  |  |  |  |  |
| **Diclofenac**  **Anti inflammatory** | O.D. (1) | O.D. (2) | O.D.  (3) | Average | % |
| 5 | 0.814 | 0.87 | 0.75 | 0.81 | 32.98 |
| 10 | 0.789 | 0.66 | 0.65 | 0.69 | 38.4 |
| 15 | 0.3 | 0.27 | 0.3 | 0.29 | 71.7 |
| 20 | 0.21 | 0.26 | 0.21 | 0.22 | 79.42 |
| 25 | 0.16 | 0.22 | 0.15 | 0.17 | 84.55 |

Figure. 6d Antiinflammatory activity
