# Supplementary material for: Heteroatom-doped carbon dots from medicinal plants as novel biomaterials for as-use biomedical applications in comparison with synthetic drug, zaltoprofen
Source: Sci Rep. 2024 Jun 7;14:13160. doi: 10.1038/s41598-024-63700-w (PMC11161473; doi:10.1038/s41598-024-63700-w)
Supplement: Supplementary file 1 — Supplementary Information 1. [file 41598_2024_63700_MOESM1_ESM.zip › Raw data of scientific reports/Fig. 8c Cell viability.docx]

| **Samples** | **Concentrations** | **OD values (triplicate) - 24hrs** | | | | |
| --- | --- | --- | --- | --- | --- | --- |
|  |  | **1** | **2** | **3** | **Average** | **% of viability** |
| Control cells (without treatment) | | 1.32 | 1.323 | 1.323 | 1.322 | 100% |
| Etoposide  (standard drug) | 50 µg | 0.875 | 0.875 | 0.875 | 0.875 | 33.81 |
| Beta TC 6 | 15µg | 0.335 | 0.334 | 0.336 | 0.335 | 74.65 |
|  | 31µg | 0.427 | 0.428 | 0.427 | 0.427 | 67.70 |
|  | 62µg | 0.518 | 0.517 | 0.514 | 0.516 | 60.96 |
|  | 125µg | 0.602 | 0.608 | 0.603 | 0.604 | 54.31 |
|  | 250µg | 0.698 | 0.694 | 0.692 | 0.694 | 47.50 |
|  | 500µg | 0.724 | 0.728 | 0.741 | 0.731 | 44.70 |

Fig. 8c. Cell viability

| **Samples** | **Concentrations** | **OD values (triplicate) - 24hrs** | | | | |
| --- | --- | --- | --- | --- | --- | --- |
|  |  | **1** | **2** | **3** | **Average** | **% of viability** |
| Control cells (without treatment) | | 1.178 | 1.177 | 1.178 | 1.178 | 100% |
| Etoposide  (standard drug) | 50 µg | 0.875 | 0.875 | 0.875 | 0.875 | 25.72 |
| Neuroblastoma cells | 15µg | 0.098 | 0.098 | 0.099 | 0.098 | 91.68 |
|  | 31µg | 0.156 | 0.158 | 0.159 | 0.157 | 86.67 |
|  | 62µg | 0.225 | 0.224 | 0.225 | 0.224 | 80.98 |
|  | 125µg | 0.337 | 0.337 | 0.337 | 0.337 | 71.39 |
|  | 250µg | 0.457 | 0.458 | 0.454 | 0.456 | 61.29 |
|  | 500µg | 0.591 | 0.593 | 0.594 | 0.592 | 49.74 |
