# Supplementary material for: Heteroatom-doped carbon dots from medicinal plants as novel biomaterials for as-use biomedical applications in comparison with synthetic drug, zaltoprofen
Source: Sci Rep. 2024 Jun 7;14:13160. doi: 10.1038/s41598-024-63700-w (PMC11161473; doi:10.1038/s41598-024-63700-w)
Supplement: Supplementary file 2 — Supplementary Information 2. [file 41598_2024_63700_MOESM2_ESM.docx]

| **S. No.** | **Phytoconstituent** | **Tests** | ***Chromolaena***  ***odorata*** | **Lantana**  **camara** | ***Moringa***  **oleifera** | ***Tridax***  **procumbens** | ***Tinospora***  **cordifolia** |
| --- | --- | --- | --- | --- | --- | --- | --- |
| 1 | Alkaloids | Mayer’s test | - | + | - | + | + |
|  |  | Dragendorff’s test | - | + | - | - | + |
| 2 | Flavonoids | Alkaline test | - | + | - | - | + |
|  |  | Conc. H_2_SO_4_ | + | + | - | + | - |
|  |  | Lead acetate test | + | + | + | + | + |
|  |  | Shinoda’s test | - | - | - | - | - |
| 3 | Sterols | Libermann-Burchard’s test | **+** | + | + | + | + |
| 4 | Terpenoids | Libermann test | - | + | - | - | + |
| 5 | Anthraquinone | Borntrager’s test | - | + | - | - | - |
| 6 | Anthocyanin | HCl Test | - | - | - | - | - |
| 7 | Proteins | Ninhydrin test | - | - | + | + | + |
|  |  | Biurettest | - | - | - | + | + |
|  |  | Xanthoproteictest | **+** | **-** | + | - | - |
| 8 | Phenolic compounds | Ferric chloride test | + | + | - | + | - |
|  |  | Gelatin test | + | - | - | + | - |
|  |  | Ellagic acid test | + | - | - | + | - |
| 9 | Quinones | Conc. HCl | - | - | - | - | - |
|  |  | Alcoholic KOH | - | - | - | - | - |
| 10 | Carbohydrates | Molisch’s test | **+** | + | + | + | + |
|  |  | Fehling’s test | **+** | + | + | + | + |
| 11 | Tannins | Braymer’s test | - | + | + | + | - |
|  |  | Gelatin test | - | + | + | + | - |
|  |  | 10% NaOH test | - | + | + | + | - |
| 12 | Saponins | Foam test | + | + | + | - | + |
| 13 | Cardiac glycosides | Baljet test | - | + | + | - | + |
|  |  | Bromine water test | - | + | - | - | + |
|  |  | Keller-killani test | - | - | + | - | + |
| 14 | Glycosides | Borntrager’s test | - | - | - | - | - |
|  |  | Aq. NaOH test | - | - | - | - | - |
| 15 | Lignin | Labat test | - | - | - | - | - |
| 16 | Coumarins | Fluorescence test | - | - | - | - | - |
|  |  | NaOH test | - | - | - | - | - |
| 17 | Volatile oils | Fluorescence test | - | - | - | - | - |

**Table S1.**Phytochemical screening of *Chromolaena*, *Lantana, Moringa, Tridax and Tinospora* leaf extracts

| **** | **** |
| --- | --- |

**Fig. S1aTEM analysis of FN-CDs at 20 nm and 10 nm scale**

| **** | **** |
| --- | --- |

**Fig. S1b TEM analysis of Z- FN-CDs at 20 nm and 10 nm scale**

**Fig. S2a Optical band gap of 0.72 eV for FN-CDs**

**Fig. S2b Optical band gap of 0.32 eV for Z-FN-CDs**

| 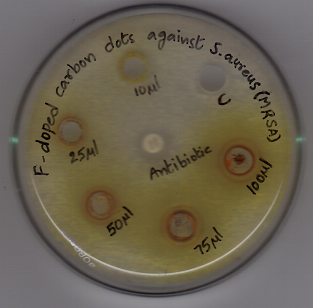 | 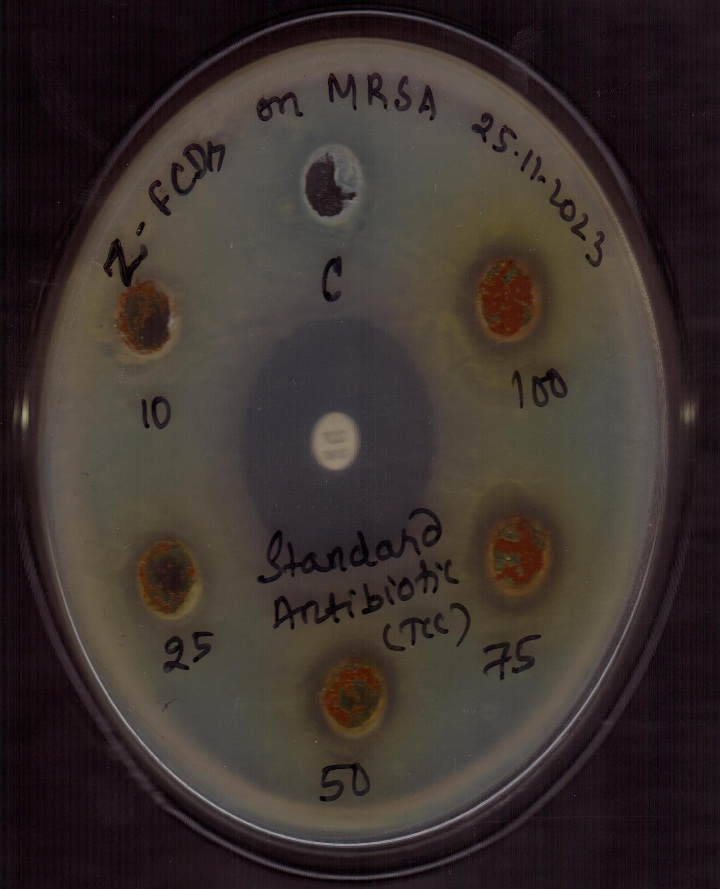 |
| --- | --- |
| 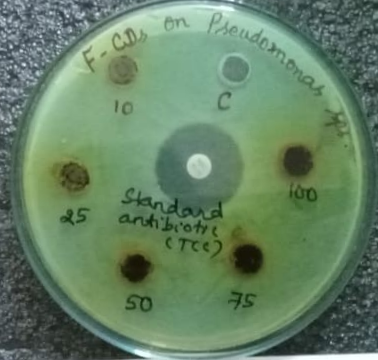 | 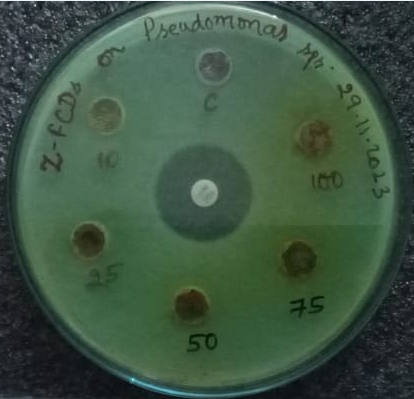 |

**Fig. S3 Antimicrobial activity of FN-CDsand Z-FN-CDs against MRSA and *Pseudomonas***
